# Remote coupling of electrical and mechanical cues by diurnal photothermal irradiation synergistically promotes bone regeneration

**DOI:** 10.1186/s12951-024-02671-6

**Published:** 2024-07-11

**Authors:** Haoqi Lei, Jiwei Sun, Zhiyin Dai, Keqi Wo, Junyuan Zhang, Yifan Wang, Baoying Zhao, Wenjie Fan, Jinyu Wang, Yunsong Shi, Cheng Yang, Bin Su, Zhiqiang Luo, Junjie Wu, Lili Chen, Yingying Chu

**Affiliations:** 1 Department of Stomatology, Union Hospital, Tongji Medical College, Huazhong University of Science and Technology, 1277 Jiefang Avenue, Wuhan, 430022 China; 2 School of Stomatology, Tongji Medical College, Huazhong University of Science and Technology, Wuhan, 430030 China; 3 Hubei Province Key Laboratory of Oral and Maxillofacial Development and Regeneration, Wuhan, 430022 China; 4 School of Chemistry, Chemical Engineering and Life Sciences, Wuhan University of Technology, Wuhan, 430070 China; 5 State Key Laboratory of Materials Processing and Die & Mould Technology, School of Materials Science and Engineering, Huazhong University of Science and Technology, Wuhan, 430074 China; 6 National Engineering Research Center for Nanomedicine, College of Life Science and Technology, Huazhong University of Science and Technology, Wuhan, 430074 China; 7 Department of Orthodontics, School of Stomatology, Air Force Medical University, Xi’An, 710032 China

**Keywords:** Electro-bioactive composite membranes, Remote tuning of electrical cues, Mechano-electrical coupling for cellular modulation

## Abstract

Recapitulating the natural extracellular physical microenvironment has emerged as a promising method for tissue regeneration, as multiple physical interventions, including ultrasound, thermal and electrical therapy, have shown great potential. However, simultaneous coupling of multiple physical cues to highly bio-mimick natural characteristics for improved tissue regeneration still remains formidable. Coupling of intrinsic electrical and mechanical cues has been regarded as an effective way to modulate tissue repair. Nevertheless, precise and convenient manipulation on coupling of mechano-electrical signals within extracellular environment to facilitate tissue regeneration remains challengeable. Herein, a photothermal-sensitive piezoelectric membrane was designed for simultaneous integration of electrical and mechanical signals in response to NIR irradiation. The high-performance mechano-electrical coupling under NIR exposure synergistically triggered the promotion of osteogenic differentiation of stem cells and enhances bone defect regeneration by increasing cellular mechanical sensing, attachment, spreading and cytoskeleton remodeling. This study highlights the coupling of mechanical signals and electrical cues for modulation of osteogenesis, and sheds light on alternative bone tissue engineering therapies with multiple integrated physical cues for tissue repair.

**Graphical Abstract:**

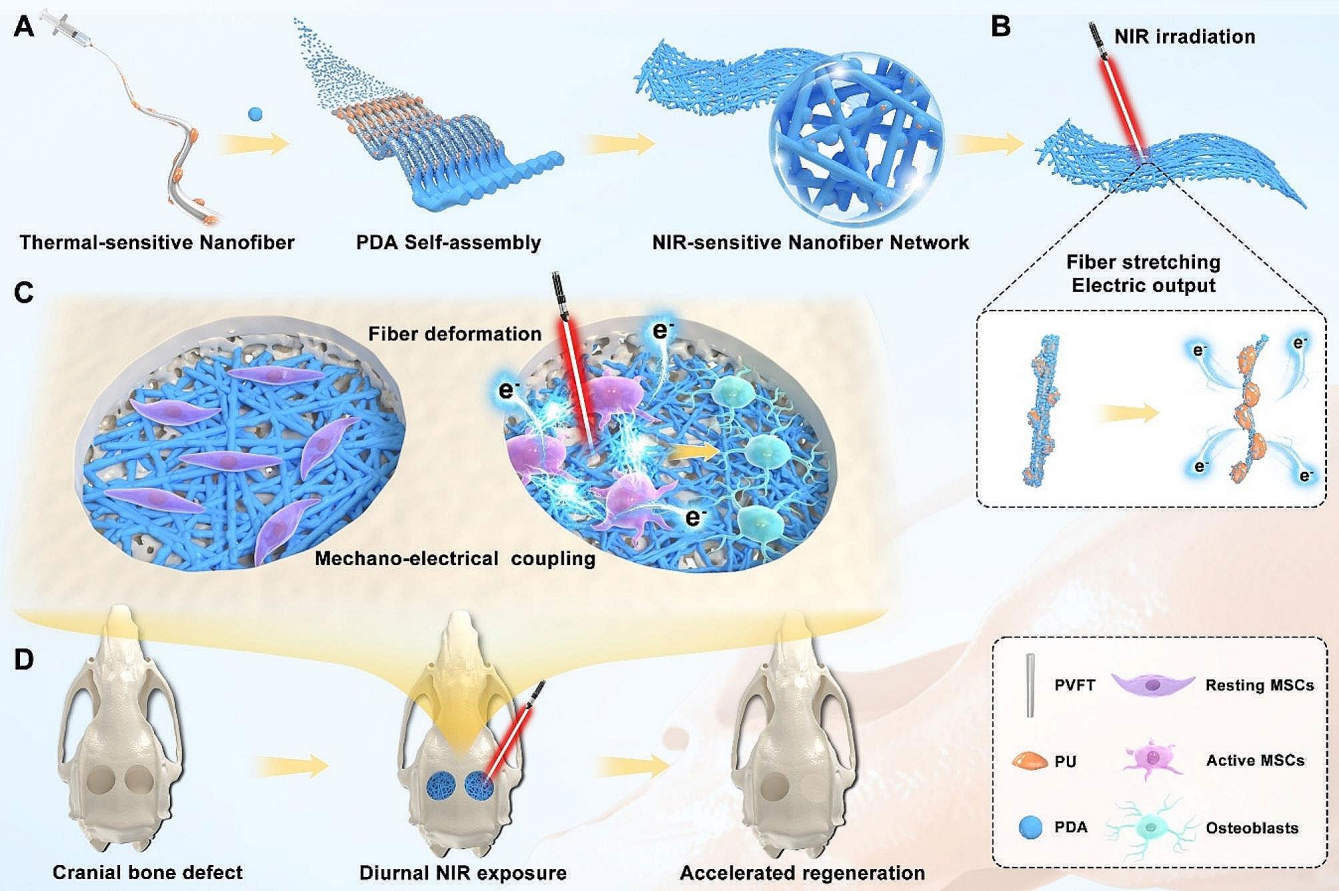

**Supplementary Information:**

The online version contains supplementary material available at 10.1186/s12951-024-02671-6.

## Introduction

Along with the increasing patients suffering from bone loss and insufficient bone regeneration achieved by clinical bone implants, the clinical demand for implanted biomaterials is in urgent need [1–4]. Natural biophysical microenvironment comprised of temperature, light, topographic structure, electrical and mechanical cues, play crucial roles in homeostasis of normal tissue and regeneration of defects and wounds [5–8]. In particular, mechanical and electrical cues are of great importance and have been the focus of research during recent years [9, 10]. Based on this theory, novel therapeutic strategies in tissue engineering have been developed to mimic extracellular characteristics for improved tissue regeneration [11, 12]. Extensive studies have revealed that simulating endogenous physical cues of extracellular matrix could modulate cell functions [13–15]. Indeed, endogenous mechanical and electric properties have attracted great attention, as they have been discovered to modulate various mesenchymal cell behaviors [16–18], and external mechanical or electrical stimulation has been broadly studied and proved to facilitate tissue regeneration including bone, cartilage, nerve and skin [19–21]. Implantable polymer membrane materials with electrical cues have exhibited significant application experience in tissue engineering and regenerative medicine due to their significant enhancement in MSC cell metabolism and tissue differentiation [22, 23]. Biomaterials inspired by mechanical cues have also made a number of advances due to their ability to act directly on the cellular behavior [24, 25]. YAP (yes-associated protein), an endotranscriptional effector of the Hippo signaling pathway, has been identified to sense a variety of mechanistic signals within the extracellular environment [26]. Recent studies have confirmed that there is a crosstalk among YAP and bone metabolic pathways such as Wnt/β-catenin, which could affect osteogenic differentiation [27]. However, conventional invasive mechanical/electrical stimulating devices in clinical practice have a narrow range of adjustable parameters and low adjustable precision, resulting in unsatisfactory clinical treatment results, as well as potential risk of infection and secondary surgical injuries [28, 29]. Accurate dosage control and precise location of working area is also difficult achieve [30, 31]. Thus, supplementation of biophysical mechanical and electrical signals at natural level with implanted biomaterials is regarded as alternative method for optimal bone regeneration. Biomaterials such as elastic hydrogels or electroactive nanoparticles could provide mechanical or electrical cues separately [32, 33], but still failed to acquire complete satisfactory outcomes. Therefore, mechano-electrical coupling is expected to produce synergistic effect, exhibiting substantial potential for high-performance tissue regeneration.

Within the native microenvironment, diurnal variations in electrical and mechanical signals are critical for modulation of cellular functions, and they are accompanied along with each other [34–36]. Hence, simultaneous and mutual coupling of mechanical and electrical cues via remote control and regulation for their diurnal shifting is of significant importance for facilitated bone regeneration. In the natural extracellular microenvironment, mesenchymal stem cells could on-demand regulate electric field via mechanical deformation of matrix fibers [37–39]. This natural phenomenon inspires our concept that remote modulation of piezoelectric fibers could simultaneously combine and control mechanical and electrical cues, thereby efficiently providing a local mechano-electrical microenvironment. However, there still lacks a convenient and efficient strategy to simultaneous mechano-electrical coupling in response to remote tuning.

Here, Near-infrared (NIR) mediated photothermal effect was selected for remote control. we first fabricated the flexible membranes consisting of piezoelectric fibers by employing electrospun poly (vinylidene fluoridetrifluoroethylene) nanofiber [PVFT NF]. To achieve remote control of fiber deformation, polyurethane (PU) serves as highly elastic phase change polymer (HEPCP) and grows inside the interspace among PVFT NFs. The nanocomposite 3D framework was then encapsulated by photothermal-sensitive agent polydopamine (PDA). Photothermal effect of PDA in response to NIR irradiation subsequently induced volume variation of PU due to its microcrystallization phase transition. The expansion and shrinkage of PU mediates elastic deformation of PVFT NFs, driving excellent piezoelectric effects of PVFT NFs. The bi-directional shape changes of PVFT NFs (deformation and restoration) simultaneously generated mechanical and electrical signals in response to NIR irradiation. By optimizing the cyclic NIR irradiation strategy, high-performance diurnal mechano-electrical coupling was successfully accomplished. Based on the results from osteogenic differentiation measurement and RNA sequencing of in vitro cells co-cultured with nanocomposite membranes, as well as intervention on cranial bone defects in rats for up to 8 weeks using cyclic NIR therapy in vivo, the biomimetic mechano-electrical orchestration could significantly promote bone regeneration within defect region, and this outcome was attributed to enhanced osteogenic differentiation of bone marrow stem cells (BMSCs). The novel remote tuning strategy contributed to coupling and diurnal shift of mechanical and electrical signals, which significantly promoted osteogenesis, and represents a promising tool for recapitulating synergistic mechano-electrical microenvironment during tissue regeneration.

## Results and discussions

### Fabrication and characterization of NIR-responsive piezoelectric nanocomposite membrane

**Fig. 1:**
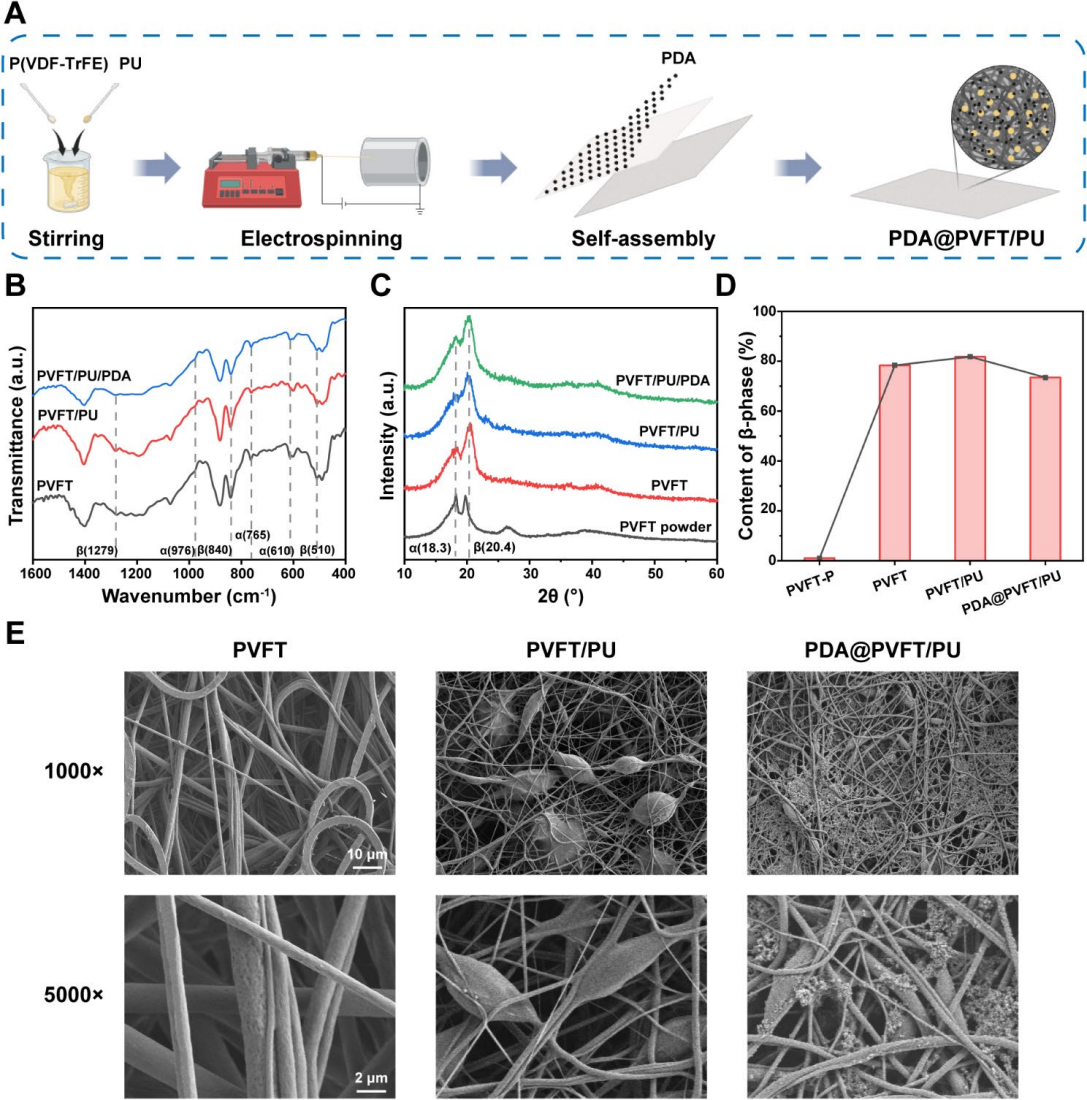
Fabrication and structural characterization of the NIR-responsive nanocomposite membrane. (**A**) Schematic diagram depicting the fabrication of PDA@PVFT/PU nanocomposite membrane. Created by BioRender online tool. (**B**) FTIR spectrum of PVFT, PVFT/PU and PDA@PVFT/PU nanocomposite membrane. (**C**) XRD patterns of PVFT, PVFT/PU and PDA@PVFT/PU nanocomposite membrane. (**D**) Comparison of the content of β-phase of PVFT powder and nanocomposite membranes. (**E**) SEM images of PVFT, PVFT/PU and PDA@PVFT/PU nanocomposite membranes (Scale size = 10 μm and 2 µm)

PDA@PVFT/PU piezoelectric nanocomposite membranes were constructed by electrospinning and surface self-assembly (Fig. 1A). In detail, PU was synthesized (Figure S1A) and uniformly distributed within the PVFT electrospinning solution. After stretching and polarization by high-voltage electric field and surface self-assembly in the PDA polymerization solution (Figure S1B), PU particles were uniformly embedded into PVFT NF matrix with encapsulation of PDA nano-particles on their surface. The phase structure of nanocomposite membranes was analyzed by Fourier transform infrared (FTIR), Attenuated Total Refraction (ATR) and X-ray diffraction (XRD) spectroscopy. There are two crystalline phases (α and β) in PVFT NFs, of which α phase is the most common and stable nonpolar phase, while the more β content accounts for the stronger piezoelectric properties [40]. FTIR and ATR shows three β-phase correlated absorption bands at 1279, 840 and 510 cm^− 1^(Fig. 1B, Figure S3). In XRD, the main characteristic peaks of the α and β phases of the membranes appeared at 18.3° and 20.4° (Fig. 1C). The relative amount of the β-phase was calculated using the absorption intensities of the β-phase at 840 cm^− 1^ and the α-phase at 765 cm^− 1^. According to the equation: [41]

$${\rm{F}}\left( {\rm{\beta }} \right){\rm{ = }}{{\rm{A}}_{\rm{\beta }}}{\rm{/}}\left( {{\rm{1}}{\rm{.3}}{{\rm{A}}_{\rm{\alpha }}}{\rm{ + }}{{\rm{A}}_{\rm{\beta }}}} \right){\rm{100\% }}$$

F(β) denotes the β-phase content and Aα and Aβ are the absorbance at 765 cm^− 1^and 840 cm^− 1^. The calculated β-phase content of the PVFT membranes were 79%, confirming its high-voltage electrical response. Due to the viscosity of the electrospinning solution after the addition of PU, the diameter of nanofibers in the PVFT/PU nanocomposite membranes was finer, which led to a better polarization effect within the high electric field. So, the proportion of β-phase in PVFT/PU nanocomposite membranes is slightly increased to 81% [42]. Similarly, the ratio of β-phase is slightly reduced in PDA@PVFT/PU nanocomposite membranes due to the hydrogen bonding between -OH, -NH2 and PVFT NFs with PDA (Fig. 1D). In summary, XRD and FTIR analysis confirmed the superior piezoelectric behavior of the nanocomposite membranes.

Subsequently, Scanning Electron Microscopy (SEM) was used to visualize the PVFT NFs and particle morphology (Fig. 1E). PVFT is a porous 3D network structure constructed by nano-scale fibers. SEM images of PVFT/PU nanocomposite membranes showed that PU particles were uniformly dispersed and tightly contacted with the PVFT NFs due to the blended electrospinning of PVFT and PU polymers. Compared with PVFT/PU nanocomposite membranes, the PU and fiber network from PDA@PVFT/PU nanocomposite membranes contained a large number of PDA nanoparticles filling and coating.

The nanocomposite membranes possessed good hydrophilicity and flexibility. By evaluating the water contact angles on the surface of the nanocomposite membrane, it was confirmed that the addition of hydrophilic PDA could render the nanocomposite membrane optimal hydrophilicity (Figure S4). The tensile strength of PVFT membranes was within the range of 20–25 MPa, and the strain at break reaches 178%. It could be observed that the decrease in mechanical strength due to the thinning of fibers by adding PU and the increase in mechanical strength due to the addition of PDA are almost offset, and the flexibility of PDA@PVFT/PU nanocomposite membrane was suitable for clinical application (Figure S5).

### NIR irradiation triggers mechanical output of PVFT NFs

**Fig. 2:**
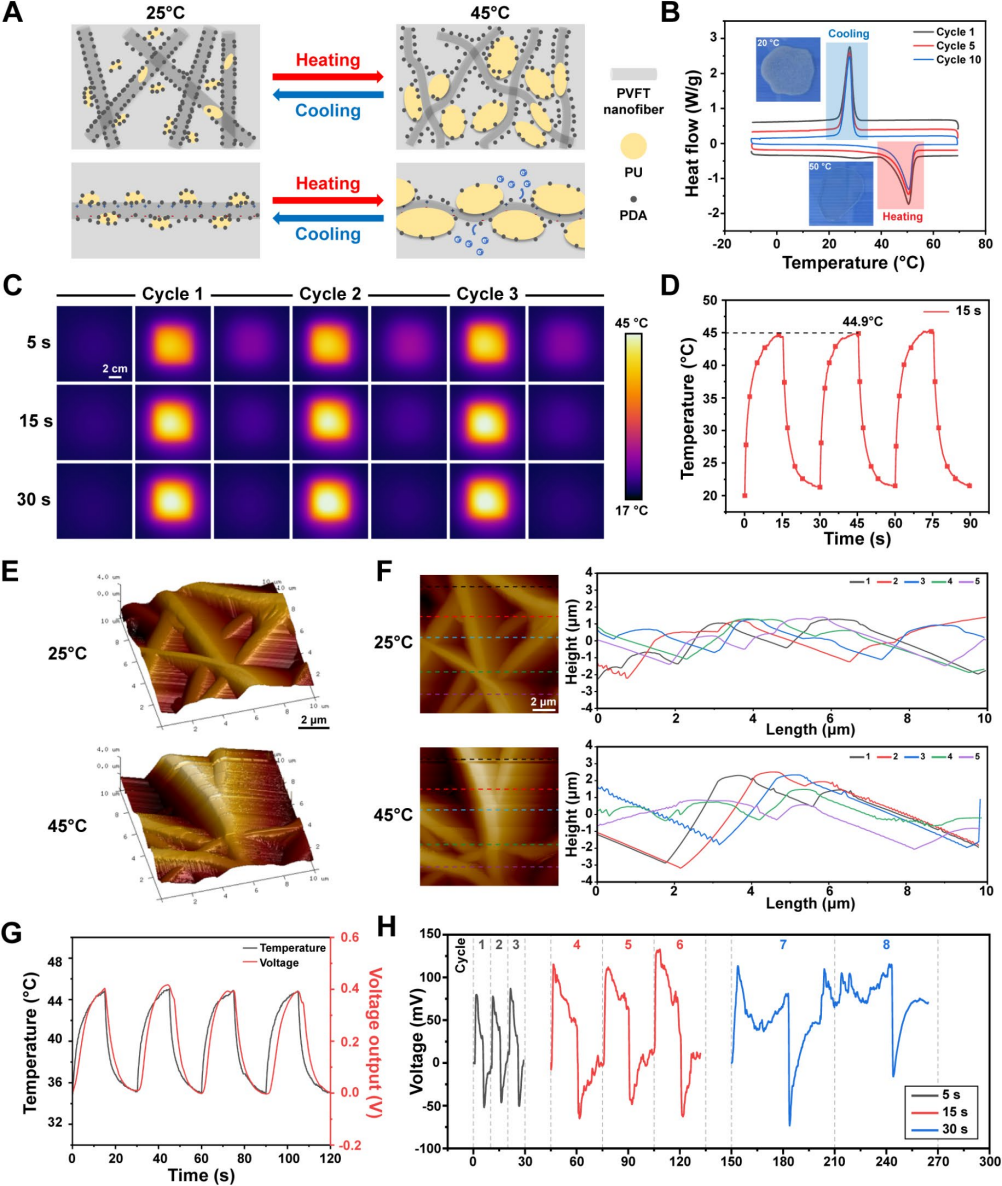
Mechano-electrical coupling properties of the NIR-responsive nanocomposite membrane. (**A**) Schematic illustration of mechano-electrical coupling mechanism. (**B**) DSC curves of PU in 10 heating-cooling cycles. (**C**) Thermal infrared photograph of PDA@PVFT/PU nanocomposite membranes at different NIR irradiation cycles (Scale size = 2 cm). (**D**) Temperature variations of PDA@PVFT/PU nanocomposite membranes within 15s NIR irradiation cycle. (**E**-**F**) AFM images and analysis of surface height of the PVFT NFs inside PDA@PVFT/PU nanocomposite membranes during NIR irradiation cycle (Scale bar = 2 μm). (**G**) Voltage output evaluation of PDA@PVFT/PU nanocomposite membrane during controlled temperature variations. (H) Real-time voltage tracking of PDA@PVFT/PU nanocomposite membrane for different NIR irradiation cycles (5 s, 15 s and 30 s)

Upon NIR irradiation on the PDA@PVFT/PU nanocomposite membranes, the temperature rapidly rises to a certain high temperature due to the photothermal effect of the surface PDA particles [43, 44]. The PU microcrystals, as HEPCP, in the nanocomposite membranes starts to melt, and the melting of the microcrystals is accompanied by volume expansion [45–48]. The volume expansion of the uniformly distributed microcrystals produces extrusion stress and internal stress on the PVFT micro-nanofibers therein, causing local deformation of the PVFT fibers. When the NIR irradiation is turned off, the temperature of the composite membrane decreases rapidly. During the cooling process, the PU microcrystals gradually crystallize, accompanied by volume contraction, so that the internal stress and external extrusion stress of the fiber disappears, and the fiber deforms in the reverse direction to restore its original shape. In summary, the mechanical response is achieved by the fiber deformation induced by the reversible phase transition of PU microcrystals upon NIR irradiation, which results in the NIR-responsive mechanical output of the nanocomposite membrane (Fig. 2A). Differential scanning calorimetry (DSC) images showed that the phase transition temperature of nanocomposite membrane is stable at 42.62–42.99℃, a temperature that effectively promotes osteogenesis through thermal effects [49–52], which was suitable for its potential application in medical treatment (Fig. 2B, Figure S6, S7). As detected by the thermal sensor, the PDA@PVFT/PU nanocomposite membrane has a good photothermal ability to rapidly change the temperature under NIR irradiation cycle (Fig. 2C). The temperature of PDA@PVFT/PU nanocomposite membrane rose to a maximum of 41.0 °C during the 5-second irradiation cycle, and to 44.9 and 47.8 °C during the 15-second and 30-second cycles respectively (Fig. 2D, Figure S8). This indicated that the maximum temperature range of the 15-second cycle not only reached the lowest temperature of the phase transition, but also remained within the temperature range of osteogenic thermotherapy. That is to say, the 15-second NIR irradiation cycle was expected to achieve high-performance phase transition and medical effect.

Subsequently, Atomic Force Microscope (AFM) was used to visualize the morphologic change of NIR-stimulated PVFT NFs. When the temperature increases from room temperature to 45 °C, PVFT NFs are subjected to external extrusion and internal stresses, resulting in deformation in the transverse and longitudinal directions, which can be observed as a change in fiber orientation and altitude difference on the surface under AFM probe (Fig. 2E, Figure S9). Cross-sectional analysis of the surface height of the PDA@PVFT/PU nanocomposite leimembrane consistently showed that NIR-responsive temperature increase led to deformation of the PVFT NFs within the composite membranes, which could generate mechanical signal and piezoelectric output simultaneously (Fig. 2F).

### Rhythmic NIR irradiation on PDA@PVFT/PU membrane triggers mechano-electric coupling

To further verify the effect of NIR-induced electric output, we perform electric output testing for the nanocomposite membranes (Figure S10). Piezoelectric output testing of the membranes corroborates the β-phase results obtained in the previous section. In particular, PDA@PVFT/PU nanocomposite membrane could produce a voltage of 1.0-1.2 v at the surface pressure of 5 N, which indicated its excellent piezoelectric property (Figure S11). According to the previous section, the NIR-responsive temperature variations cause PVFT NFs deformation and produce mechanical outputs. PVFT NFs, as highly efficient piezoelectric fibers, were expected to produce cyclic piezoelectric outputs during the rhythmic deformation process, leading to a diurnal mechano-electric coupling output (Fig. 2A). By detecting the absolute voltage output of the PDA@PVFT/PU nanocomposite membrane along with temperature variations, we determined that the diurnal mechano-electric output was successfully achieved (Fig. 2G). According to the different NIR irradiation cycles proved above, we performed real-time monitoring for the piezoelectric output of the composite film. The 5 s and 15 s irradiation cycles showed stable cyclic piezoelectric output during both warming up and cooling down, and the output voltage of the 15 s cycle can reach up to 130 mV. However, the piezoelectric output of the 30 s is not regular, and its highest output voltage is only 120 mV (Fig. 2H). We then hypothesized that, when the period of PU microcrystal melting phase transition within the nanocomposite membrane is longer than the period of the NIR-responsive temperature variations, the NIR-responsive temperature cycle can match up with the PU microcrystal melting phase transition period. It is shown as the longer time the temperature changes, the longer time for the PU microcrystal phase transition to reach larger amplitude, and the larger the piezoelectric output of the PVFT NFs, which is compatible with the results reflected by the 5 s and 15 s irradiation cycles. When the period of NIR-responsive temperature change is longer than the melting phase transition of PU microcrystals, it would cause the PU microcrystals to directly phase into a molten state during the first temperature cycle, which will greatly reduce the stress of PVFT NFs and result in irregular output voltage such as 30 s irradiation cycles. Also, the maximum temperature reached in a 15 s NIR cycle is within 42–45 °C, which is clinically optimal for thermotherapy to promote osteogenesis. In summary, 15 s NIR irradiation model could perform efficient mechano-electrical coupling and showed substantial potential in regulation of stem cell function and tissue engineering.

### Evaluation of osteogenic differentiation of BMSCs on PDA@PVFT/PU in vitro

**Fig. 3:**
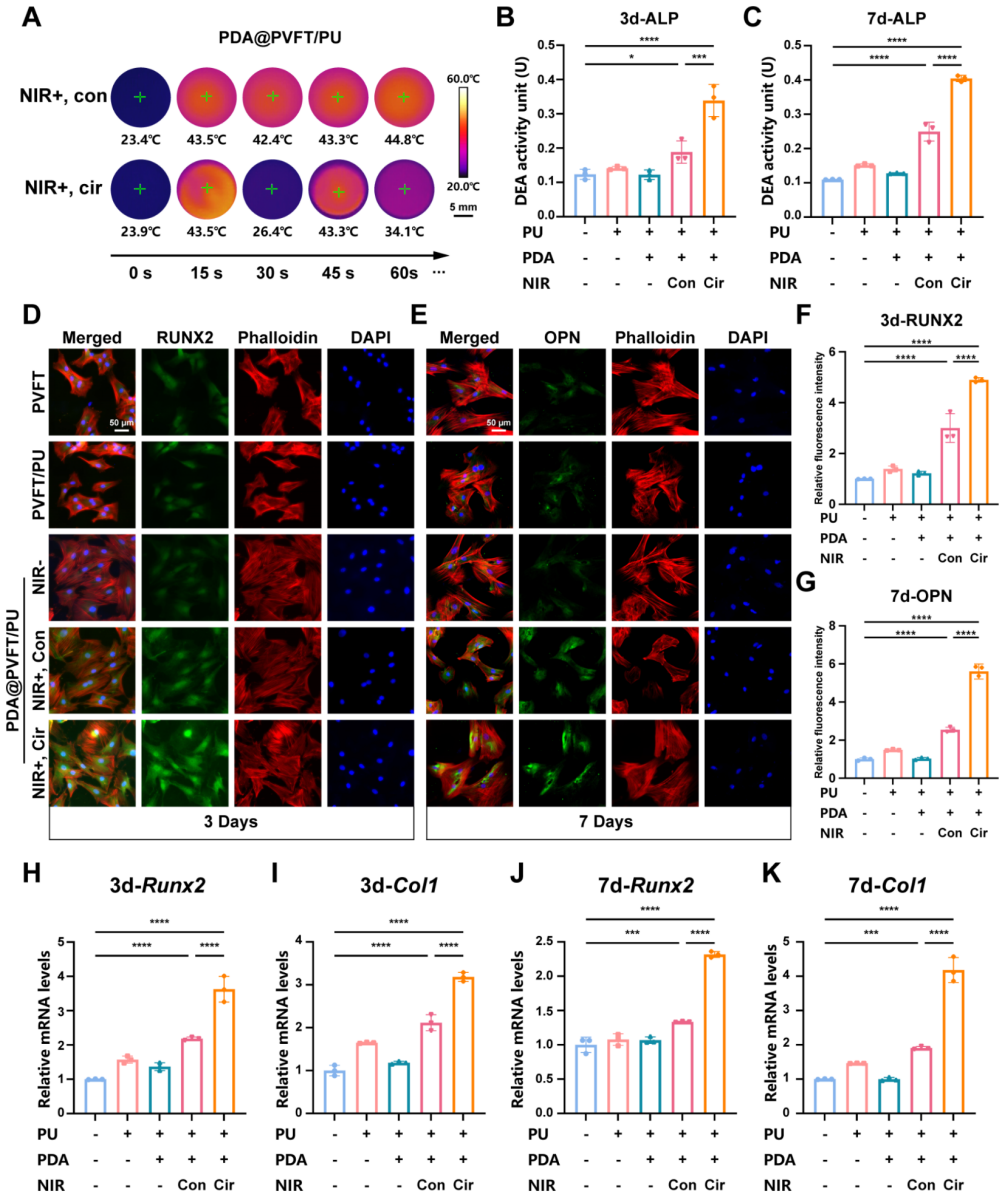
Diurnal NIR exposure enhances osteogenic differentiation of BMSCs *in vitro.* (**A**) Control of temperature variations in 24-well plate under continuous NIR (NIR+, Con) and circular NIR (NIR+, Cir) irradiation. NIR power = 0.5 W/cm^2^. Scale bar = 5 mm (**B**) and (**C**) The ALP activity of BMSCs under continuous and circular NIR irradiation in nanocomposite membranes after 3 and 7 days co-culture. (**D**) and (**E**) Representative immunostaining images of 3-day RUNX2 (green), 7-day OPN (green), Phalloidin (red) and cell nuclei (DAPI, blue) of BMSCs cultured under indicated conditions for 3 and 7 days (Scale size = 50 μm). (**F**) and (**G**) Quantitative analysis of fluorescence intensity of 3-day RUNX2 and 7-day OPN. (**H**), (**I**), (**J**) and (**K**) The expression of osteo-related genes, *Runx2* and *Col1* at day 3 and 7 respectively. *p < 0.05, **p < 0.01, ***p < 0.001 and ****p < 0.0001 (*n* ≥ 3 per group)

In order to characterize the effect of the diurnal mechano-electrical coupling output of the circular NIR response on osteogenic efficacy, we grouped the components with NIR irradiation into continuous NIR (NIR+, Con) and circular NIR (NIR+, Cir). Thermal imaging showed that NIR irradiation of cell culture plates from both groups resulted in maximum temperatures in the range of 42–45 °C with stable temperature cycling at the power of 0.5 W/cm^2^ (Fig. 3A). The cell counting kit 8 (CCK8) assay and live/dead cell staining were performed to assess the biocompatibility of nanocomposite membranes, and results suggested that the nanocomposite membranes are appropriate and safe for cell survival (Figure S12A-B). Alkaline phosphatase (ALP) assay revealed the highest ALP activity of BMSCs in PDA@PVFT/PU (NIR+, Con) group after 3 and 7 days of culture (Fig. 3B-C). These results were corroborated by Immunofluorescence staining, with significantly upregulated RUNX2 after 3 days (Fig. 3D, F) and OPN after 7 days (Fig. 3E, G). Furthermore, the expression of osteogenic-related marker genes *Runx2*, *Col1a1*, *Alp* and *Osx* were analyzed by real-time quantitative Polymerase Chain Reaction (RT-qPCR) after 3 and 7 days of BMSCs culture. All these osteogenic genes were substantial increased in PDA@PVFT/PU (NIR+, Con) group (Fig. 3H-K, Figure S15). As markers of early stage of osteogenic differentiation, the expression of RUNX2 and ALP were additionally analyzed by western blot after 7 days of culture. Images and the analysis results showed that all these osteogenic proteins were substantially increased in PDA@PVFT/PU (NIR+, Con) group, which was consistent with the previous assays (Figure S16). Specifically speaking, high-performance diurnal mechano-electrical coupling of PDA@PVFT/PU triggered by circular NIR irradiation substantially promoted osteogenic differentiation compared with other groups in vitro.

To explore the level of matrix mineralization in BMSCs at late stage of osteogenic differentiation, the expression of Collagen I (COL1A1) and Osteocalcin (OCN) have been further determined. Secretion and maturation of Col1A1 is the basis for mineral deposition and matrix mineralization in the late stage of osteogenesis [53, 54]. Meanwhile, OCN is a highly abundant bone protein which is the key to regulate mineral deposition during bone remodeling [55, 56]. Western blot assays showed the highest expression of COL1 and OCN in PDA@PVFT/PU (NIR+, Cir) group in vitro, indicating that circulating mechano-electrical coupling stimulation resulted in obviously increased levels of matrix mineralization in vitro (Figure S17), which was consistent with previous results of immunofluorescence of COL1A1 and OCN after 7 and 14 days (Figure S13-14). Together, the above results regarding COL1 and OCN expression indicated that diurnal mechano-electrical coupling of PDA@PVFT/PU promoted cellular matrix mineralization in vitro.

Finally, to verify whether NIR-responsive mechano-electrical coupling has an effect on osteoclasts during bone regeneration in vitro, we seeded monocytes in nanocomposite membranes and induced osteoblast differentiation with or without circular NIR irradiation. After 10 days of osteoclast differentiation induction, the intensity of the osteoclast marker tartrate resistant acid phosphatase (TRAP) showed no statistically significant difference in TRAP activity of osteoblasts in each group (Figure S18), suggesting that diurnal mechano-electrical coupling had no significant effect or impact on osteoblast differentiation.

Herein, these results indicated that PDA@PVFT/PU nanocomposite membrane with circular NIR irradiation showed superior inductivity for osteogenic differentiation and matrix mineralization of BMSCs by diurnal mechano-electrical coupling output.

### High-performance diurnal mechano-electrical coupling of PDA@PVFT/PU promote osteogenic differentiation in vitro

**Fig. 4:**
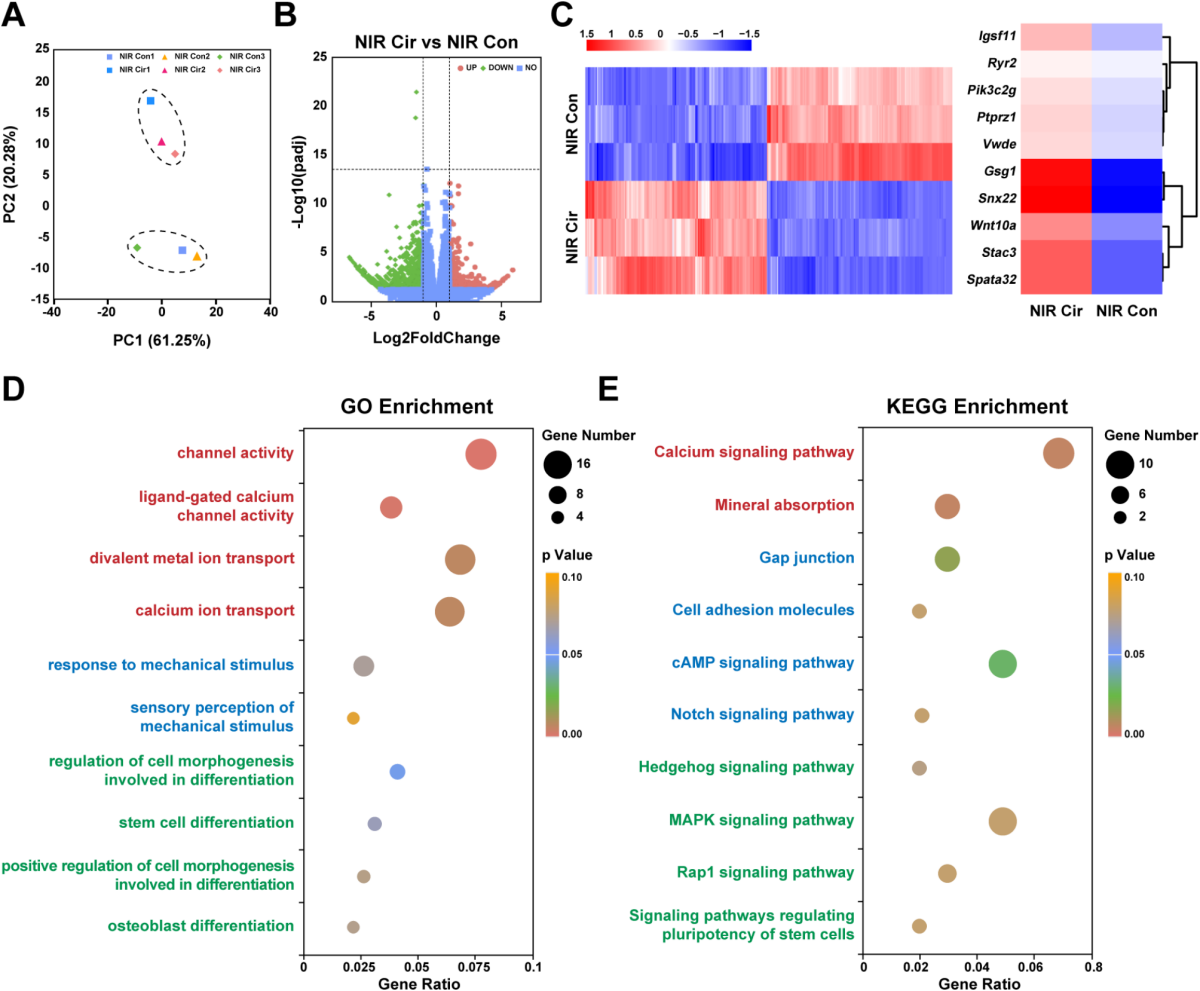
Mechanical and electrical cues are closely associated with rhythmic NIR-induced osteogenesis. **(A)** Principal Component Analysis (PCA) of the RNA sequencing profile of BMSCs after 7-day culture in continuous and circular NIR irradiation. (**B**) Volcano plot of differentially expressed genes from RNA-seq analysis performed on BMSCs cultured in continuous and circular NIR irradiation after 7 days. (**C**) The heatmap of differentially expressed genes from RNA sequencing. (**D**) Gene ontology (GO) enrichment analysis of the upregulated genes in BMSCs cultured in circular NIR irradiation after 7 days. (**E**) Kyoto Encyclopedia of Genes and Genomes (KEGG) analysis of upregulated genes in BMSCs cultured in circular NIR irradiation after 7 days

To elucidate cellular response to circular NIR irradiation on PDA@PVFT/PU on the transcriptomic scale, RNA sequencing (RNA-seq) was performed to analyze differential gene expression of BMSCs harvested from the PDA@PVFT/PU (NIR+, Con) group and PDA@PVFT/PU (NIR+, Cir) group after 7-day culture. Principal component analysis (PCA) revealed a clear separation between cells cultured in NIR Con group and NIR Cir group (Fig. 4A). When compared with cells in continuous NIR irradiation, 1256 DEGs were identified from cells cultured in circular NIR irradiation (Fig. 4B-C). Top ten DEGs were “*Gsg1*”, “*Ptprz1*”, “*Stac3*”, “*Pik3c2g*”, “*Wnt10a*”, “*Spata32*”, “*Ryr2*”, “*Snx22*”, “*Igsf11*”, “*Vwde*”, the functions of which ranging from cell metabolism, calcium binding, actin binding, promotion of cell adhesion and osteogenic differentiation. Among them, “*Wnt10a*” and “*Snx22*” are osteogenic-related genes. As one of the Wnt gene family, activation of *Wnt10a* promoted osteogenesis-related pathways in BMSCs [57]. As one of the genes encoding peptidylprolyl isomerase B (PPIB), *Snx22* downregulation was associated with Osteogenesis Imperfecta, Type Ix and Brittle Bone Disorder [58]. These results indicated that it is the NIR irradiation mode but not the NIR irradiation itself that contributed to the gene expression pattern alterations of BMSCs. Gene ontology (GO) analysis determined that channel activity, divalent metal ion transport, calcium ion transport, response to mechanical stimulus as well as regulation of cell morphogenesis involved in differentiation associated genes were over-represented in the NIR Cir group (Fig. 4D), suggesting the transport of divalent metal cations such as calcium ions, mechanical stimulation to cells, and enhanced differentiation of stem cells due to various causes may be the potential mechanism in osteogenic differentiation after circular NIR irradiation. Enriched KEGG pathways such as the calcium signaling pathway, gap junction, cAMP signaling pathway, and MAPK signaling pathway confirming the previous results (Fig. 4E).

**Fig. 5:**
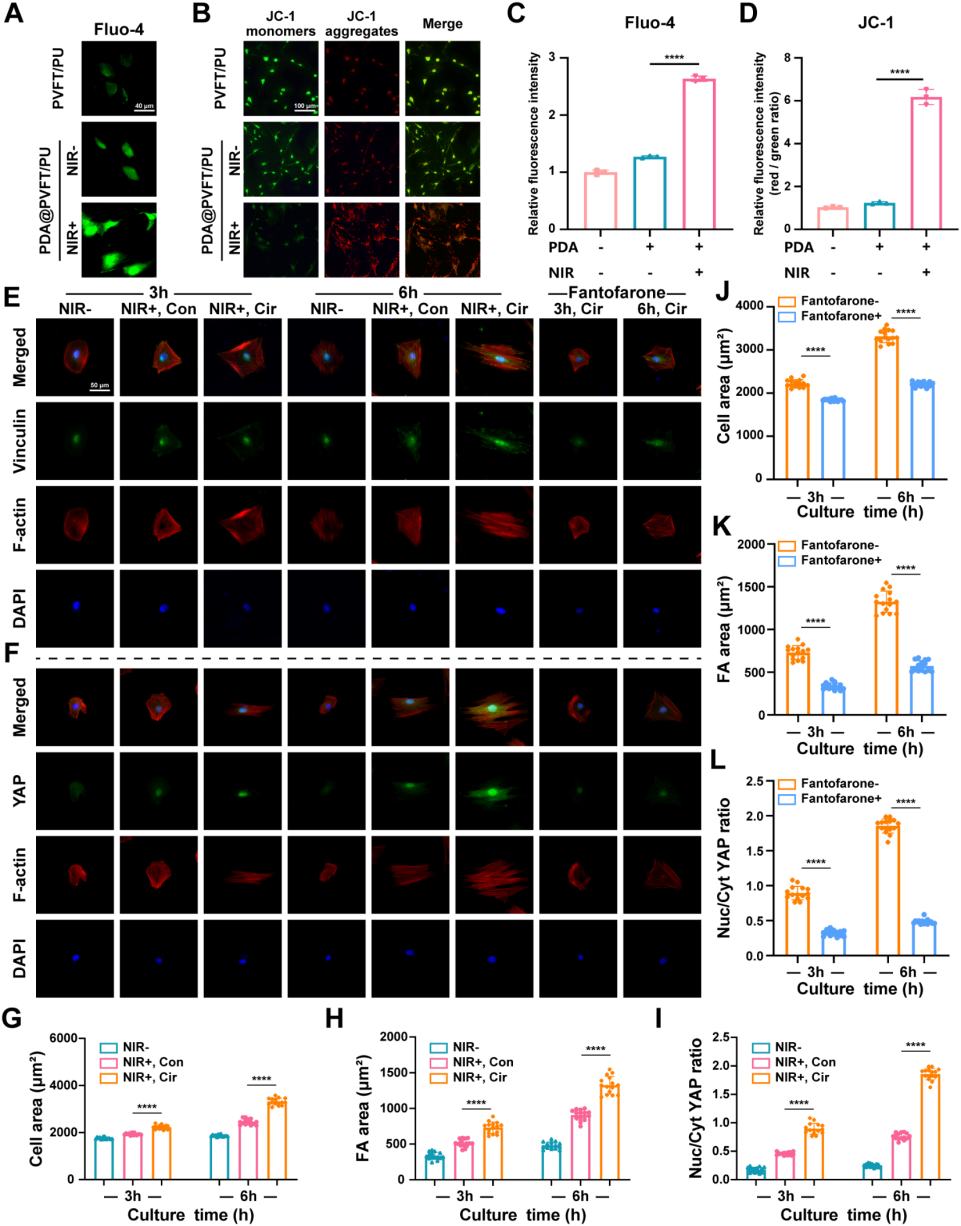
NIR-mediated physiological electric output facilitates osteogenesis by amplifying cellular mechanosensing. (**A**) Representative immunofluorescence images of Flou-4 (green) signals in BMSCs cultured for 24 h (Scale bar = 40 μm). (**B**) Representative immunofluorescence images of JC-1 monomers (green) and JC-1 aggregates (red) in BMSCs (Scale bar = 100 μm). (**C**) and (**D**) Quantitative analysis of relative mean fluorescence intensity of Flou-4 and JC-1 in different groups. (**E**) Representative immunofluorescent images of focal adhesions (FAs, Vinculin, green), YAP (green), actin network (Phalloidin, red), and cell nuclei (DAPI, blue) in BMSCs cultured in different NIR irradiation treatments for 3 and 6 h (Scale size = 50 μm). (**F**), (**G**) and (**H**) Quantitative analysis of the cytoskeleton area, FA spreading area and nuclear-to-cytoplasmic ratio of YAP per cell after 3 and 6 h culture. (**I**), (**J**) and (**K**) Quantitative analysis of the cytoskeleton area, FA spreading area and nuclear relative to cytoplasmic ratio of YAP per cell after the addition of calcium channel inhibitors Fantofarone for 3 and 6 h culture. *p < 0.05, **p < 0.01, ***p < 0.001 and ****p < 0.0001 (*n* ≥ 15 per group)

Based on RNA sequencing results, we conducted experiments to confirm the role of calcium signaling pathway in NIR-induced mechano-electrical coupling promoting osteogenic differentiation. It has been demonstrated that electrical stimulation induces a cellular cascade response through initiated voltage-gated calcium channels to promote bone defect repair, and related biomaterials have been commonly reported [59, 60]. To explore the results of calcium channel activation, Fluo-4 calcium assay was processed and demonstrated superior intracellular Ca^2+^ concentration of BMSCs in PDA@PVFT/PU (NIR+) group compared with the other groups after 24 h of culture (Fig. 5A and C). JC-1 is an ideal fluorescent probe widely used to detect mitochondrial membrane potential (∆Ψm) [61]. Fluorescent images and ratio of JC-1 aggregates and monomers indicated that BMSCs in PDA@PVFT/PU got higher mitochondrial membrane potential after NIR irradiation (Fig. 5B and D). As mitochondrial Ca^2+^ homeostasis is fundamental to the regulation of mitochondrial membrane potential and intracellular Ca^2+^ homeostasis, elevated intracellular Ca^2+^ concentration leads to increased uptake of Ca^2+^ by mitochondria, resulting in a higher mitochondrial membrane potential [62, 63]. Herein, these results indicated NIR-responsive electrical stimulation promotes calcium ion endocytosis and membrane potential changes.

Then, we seeded the cells in PDA@PVFT/PU and explored the mechanical stimulation effect to BMSCs caused by NIR irradiation. After 3 h and 6 h of culture, confocal microscopy showed that the BMSCs cultured in circular NIR irradiation exhibited more abundant cytoskeletal organization and significantly larger cell spreading area, as compared to the continuous NIR irradiation group (Fig. 5E and G). Furthermore, cells cultured in circular NIR irradiation developed larger focal adhesions (FAs) stained by Vinculin (Fig. 5E and H). Yes-associated protein (YAP) is a mechano-transducer of the Hippo pathway and plays an important role in mechanical transduction [64]. In response to mechanosensory regulation, increased cytoskeletal tension promotes YAP activation and nuclear translocation [65]. Positive feedback pathways are formed through the amplification of mechanical signals by YAP, which promotes F-actin remodeling and enhances the assembly of FAs complexes [66]. Meanwhile, nuclear YAP binds to Runx2 and promotes BMSC osteogenic differentiation [67, 68]. Immunofluorescence results showed that YAP was almost exclusively localized in the nuclei in PDA@PVFT/PU (NIR+, Cir) group after 3 h and 6 h of culture, which indicated superior mechanical signaling (Fig. 5F and I). These results suggested that circular NIR irradiation promoted mechanical signal sensing and transducing.

Furthermore, with inhibition of calcium channel by Fantofarone, cell spreading area and FA area of BMSCs cultured under circular NIR irradiation exhibited a significant decrease after 3 h and 6 h respectively (Fig. 5E, F, J and K). Meanwhile, there was also a significant reduction in nuclear/cytoplasmic ratio of YAP after the addition of Fantofarone for 3 h and 6 h (Fig. 5F and L). These indicated that NIR-induced enhancement in mechano-sensing was largely determined by electric signal-mediated calcium channeling. ALP assay, immunofluorescence staining and RT-qPCR showed a significant decrease in osteogenic differentiation of BMSC with the inhibition of Fantofarone (Figure S19, S20 and S21), suggesting that decreased mechanical signaling pathway activity due to calcium channel blockade reduced the osteogenic efficiency of BMSCs. As the key link between osteogenic differentiation and mechanical stimulation, calcium channels have also been studied and regarded to guide biomaterial design [69]. Thus far, the blockage of calcium ion influx attenuated the osteogenic differentiation promotion of NIR-responsive diurnal mechano-electrical coupling effect, which suggested that NIR-induced electrical stimulation enhanced cellular mechanical signaling for osteogenic differentiation through calcium channeling.

Finally, with inhibition of actin polymerization by Cytochalasin D, cellular mechano-sensing is largely blocked. ALP assay, immunofluorescent staining and RT-qPCR results showed a further decrease in osteogenic differentiation of BMSCs (Figure S19, S20 and S21), which was almost identical to the group without NIR irradiation, suggesting that the effect of circular NIR irradiation was completely counteracted. By blocking cellular mechanosensing, we demonstrated that the osteogenic differentiation promotion of PDA@PVFT/PU nanocomposite membranes with circular NIR irradiation was achieved through cellular mechanical signaling pathway. Here in, circular NIR irradiation generated diurnal mechano-electrial coupling output of PVFT nanofibers, the mechanical output promoted osteogenic differentiation through cellular mechano-sensing, while NIR-induced electrical stimulation mediated enhancement of mechanical signaling via calcium channeling, which altogether promoted osteogenic differentiation. Meanwhile, many studies have also acknowledged the relationship between calcium channel activation and mechanical signaling [70–73].

Here, we focused on revealing the primary and cascade mechanism that NIR-induced mechano-electrical coupling promotes osteogenic differentiation mainly through activation of calcium channels and mechanical signaling.

### Biomimetic mechano-electrical orchestration of PDA@PVFT/PU promote bone regeneration in vivo

**Fig. 6:**
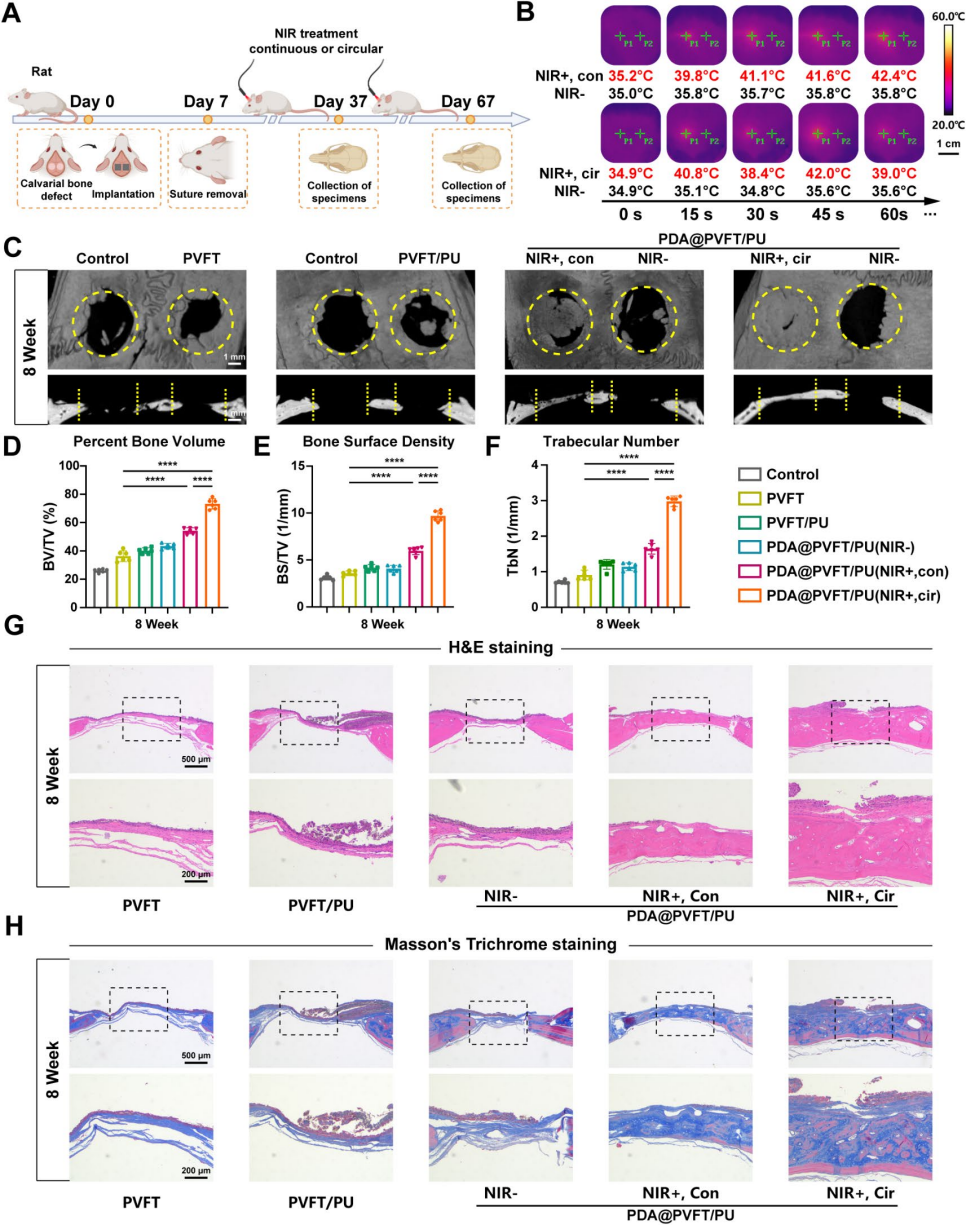
Mechano-electrical coupling promotes bone regeneration *in vivo.* (**A**)Schematic illustration of the cranial bone defect rat model and application of diurnal NIR treatment. Created by BioRender online tool. (**B**) Thermal infrared photograph of defect areas in continuous NIR (NIR+, Con) and circular NIR (NIR+, Cir) irradiation. NIR power = 0.5 W/cm^2^. Scale bar = 1 cm (**C**) Representative micro-CT images of critical-sized rat calvarial full-thickness defects after 8-week treatment in different groups. Yellow circles and dotted lines denote the boundary between new bone tissue and host bone tissue. Scale bar = 1 mm (**D**), (**E**) and (**F**) Quantitative analysis of percent bone volume (BV/TV), bone surface density (BS/TV) and Trabecular number (Tb.N). *p < 0.05, **p < 0.01, ***p < 0.001 and ****p < 0.0001 (*n* ≥ 6 per group). (**G**) and (H) H&E staining and Masson’s trichrome staining of histological sections at after 8-week irradiation treatment (Scale bar = 500 μm and 200 μm)

To further assess the osteoinductive potential of PDA@PVFT/PU with circular NIR irradiation, we prepared cranial defect models in rats (Figure S22A). Membranes were implanted covering the defects with continuous or circular NIR treatment at right side defect, and bone growth was evaluated after 4 and 8 weeks of NIR treatment (Fig. 6A). To ensure that only unilateral skull defect is irradiated, the appropriate size of the NIR laser transmitting probe was chosen to produce an NIR irradiated area of 5 mm at the appropriate distance, which is exactly the diameter of the cranial defect (Figure S22B). Thermal imaging showed that NIR treatment resulted in maximum temperatures around 42 °C with stable temperature cycling at the power of 0.5 W/cm^2^ (Fig. 6B). As evaluated by micro-computed tomography (Micro-CT), The photographs showed that more new bone formation was detected in PDA@PVFT/PU (NIR+,Cir) group after 4 and 8 weeks (Fig. 6C, Figure S23A). The bone volume fraction (BV/TV), the bone surface density (BS/TV) and the number of bone trabecula (Tb.N) in PDA@PVFT/PU (NIR+,Cir) group was significantly higher compared with those in other groups, revealing that circular NIR irradiation exhibited superior bone regeneration capability (Fig. 6D-F, Figure S23B-D). Moreover, we conducted sole thermal treatment and NIR treatment on cranial bone defects to rule out the bone regeneration effects of thermotherapy and NIR therapy. In brief, PVFT membranes were covered and treated with individual thermotherapy and NIR therapy for 4 and 8 weeks. The CT images and analysis showed a better bone regeneration than the control group, but it was still significantly lower than that from NIR-responsive mechano-electrical coupling output of PDA@PVFT/PU composite membranes (Figure S24). H&E staining showed flat and contiguous new bone formation in PDA@PVFT/PU (NIR+,Cir) group after 4 and 8 weeks (Fig. 6G, Figure S25A). Masson’s Trichrome staining confirmed the most mature osteoid tissue and abundant bone content in defect areas of the PDA@PVFT/PU (NIR+,Cir) group (Fig. 6H, Figure S25B). Meanwhile, as one of the common experimental methods for detecting mineralized nodules of osteoblasts, alizarin red staining was used to determine the level of matrix mineralization in vivo. Results confirmed the most mineralized nodules in defect areas of the PDA@PVFT/PU (NIR+, Cir) group, suggesting the highest level of matrix mineralization (Figure S28A and S29A). In addition, immunofluorescence staining against COL1A1 and OCN and immunohistochemical staining against OPN, revealed that there were the highest expression levels of PDA@PVFT/PU (NIR+,Cir) group after 4 and 8 weeks (Figure S26-27). TRAP staining confirmed an overall low level of osteoclast activity within bone defect areas, but there existed a slight increase in TRAP + cells at the margins of the bone defects in PDA@PVFT/PU groups with or without NIR irradiation (Figure S28B and S29B). This suggested that acceleration of local bone remodeling could be responsive to PDA and NIR irradiation. Collectively, these results thus verified that PDA@PVFT/PU activated by circular NIR irradiation can enhance bone regeneration though mechano-electrical orchestration in vivo.

Our NIR-responsive mechano-electrical coupling technology would be well translated for in vivo bone defect repair ranging from alveolar bone defects to long bone defects. In the bone defect scenario, we sealed the wound after implanting the NIR-responsive electroactive membrane. By triggering PDA@PVFT/PU nanocomposite membrane in the defect area through remote NIR external modulation featuring wireless and non-invasive characteristics at regular intervals daily, high-performance diurnal mechano-electrical coupling stimulation was induced, which could accelerate the pace and efficiency of bone regeneration.

## Materials and methods

### Fabrication of nanoparticles and membranes

#### Synthesis of PU

In detail, PU was synthesized by adding phenyl 4,4’-methylenebisisisocyanate (4,4’-MDI) and 1,4-butanediol (BDO) sequentially to polyethylene glycol 2000 (PEG-2000) in a two-step synthesis method (Figure S1A). First, a quantity of PEG-2000 and BDO was added to 20 mL of N, N-Dimethylformamide (DMF), and the mixture was magnetically stirred at 60 °C to dissolve. Then, 20 mL of DMF were introduced to a three-necked flask with a precise amount of 4,4’-MDI, and the mixture was continuously magnetic stirring in nitrogen at 70 °C. After the solution had been clarified, PEG-2000 solution was slowly added to it dropwise, and the reaction was carried out in nitrogen for one hour. Next, BDO solution was slowly added dropwise, and the reaction continued for 3–4 h. Finally, when the solution appears viscous, the solution is cooled to room temperature and then dried in a blast dryer to collect the PU.

### Fabrication of electrospun PVFT/PU nanofiber membranes

First, DMF and acetone were used to dissolve poly(vinylidene fluoride-co-trifluoroethylene) [P(VDF-TrFE), PVFT] powder and the produced PU. The mixture was mixed for 6 h at room temperature and sonicated for 1 h. After that, the solution was put into a 10 mL plastic syringe and fitted with a blunt-tipped, 0.2 mm stainless steel needle. The fibers were gathered on a grounded drum wrapped in aluminum foil while being pushed by a injection pump at a flow rate of 1 mm/min. The electrostatic spinning load voltages were + 12 kV and − 5 kV, the collection distance was 15 cm, and the collection drum speed was 100 rpm. After the electrostatic spinning, the membrane was put into a vacuum drying oven at 120 °C for 1 h to obtain PVFT/PU nanofiber membrane. PVFT membrane was obtained by following the appeal step without adding PU.

### Fabrication of PDA@PVFT/PU nanocomposite membranes

The dopamine poly solution was made by dissolving 2.4 g of tris-hcl in deionized water, adjusting the pH to 8 with NaOH, adding 0.4 g of dopamine hydrochloride and mixing well. PVFT/PU membrane was then immersed in the solution and shaken on a shaker at room temperature for one day. After that, deionized water was used to rinse and wash the surface of the membranes. The amount of dopamine hydrochloride was then increased to 0.8 g by repeating rinsing and washing. After dried in a vacuum oven at 120 °C for 1 h, PDA@PVFT/PU nanocomposite membranes was finally obtained.

### General characterization of nanoparticles and membranes

#### Structural characterization

The Fourier Transform Infrared Spectroscopy (FT-IR) and Attenuated Total Refraction (ATR) methods were used to confirm the chemical structures of the produced components and nanocomposite membranes. The scanning range was 4000 –400 cm^− 1^. The crystalline phase composition and material structure of the nanocomposite membranes were analyzed using XRD detection. Cu-Kα target was detected in full scan mode with scanning speed of 2°/s and detection range of 10–80°.

#### Morphology characterization

The nanocomposite membrane was treated with 75% ethanol-water solution for 10 min, then rinsed repeatedly with deionized water for 3 times and dried. The membrane was cut into squares, adhered to the electron microscope stage and plated in a vacuum plating device at 10 mA for 120 s. The surface morphology of the samples was observed using a scanning electron microscope (SEM).

#### Hydrophilicity test

Static contact angle was used to test the hydrophilicity of nanocomposite membranes at room temperature. A 5 µL drop of deionized water was placed on the surface of the membrane, and clear images was obtained by adjusting the position of the camera and the focal length of the microscope. The images were analyzed to calculate the contact angle of the nanocomposite membranes.

#### Mechanical performance evaluation

To measure the displacement of the nanocomposite membranes and the mechanical values at each instant, the membranes were sliced into rectangular specimens measuring 40 mm by 10 mm and stretched at room temperature using an electronic universal material testing equipment at a speed of 10 mm/min.

### Mechano-electrical coupling measurement of membranes

#### Phase change detection

Differential scanning calorimetry (DSC) and atomic force microscopy (AFM) techniques were used to analyze the phase transition characteristics of PU and PDA@PVFT/PU nanocomposite membranes.

DSC: Under nitrogen protection, cycles of heating and cooling were processed on PU and PDA@PVFT/PU nanocomposite membranes. The temperature range was − 10 to 70 °C.

AFM: To identify the changes of surface morphology and height brought on by temperature changing, a 1 cm^2^ square of PDA@PVFT/PU nanocomposite membrane was cut. The PDA@PVFT/PU nanocomposite membrane was then photographed at 25 °C and 45 °C under NIR irradiation.

### Photothermal performance evaluation

PDA@PVFT/PU nanocomposite membrane was placed 15 cm away from the NIR laser with power density of 0.2 mV/cm^2^.Each cycle contained of 5, 15, 30 s exposure and shielding of NIR. An infrared camera was utilized to gauge the temperature change after NIR irradiation. Temperature changes of PDA@PVFT/PU nanocomposite membrane was recorded with a thermal imaging camera.

### Piezoelectric performance testing

The composite membranes were electrically encapsulated and connected to the electrochemical workstation with wires. The piezoelectric voltage output of the composite membranes was monitored by applying 5 N mechanical stress at a frequency of 0.5 Hz.

### NIR-responsive piezoelectric performance evaluation of PDA@PVFT/PU nanocomposite membranes

In order to detect the piezoelectric output of PDA@PVFT/PU nanocomposite membrane under NIR irradiation, we constructed PDA@PVFT/PU nanocomposite membrane NIR-responsive piezoelectric detection device. On the upper and lower surfaces of the PDA@PVFT/PU nanocomposite film, a layer of copper foil was applied as electrodes with dimensions of 18*18*0.16 mm^3^, holes with dimensions of 80*80 mm^2^ were carved into the copper foil, and conductive copper wires were attached to the copper foils on both sides. NIR irradiation was then applied to the portion of the membrane exposed in the skeletonized holes.

PDA@PVFT/PU nanocomposite membrane NIR-responsive piezoelectric detection device was connected to electrochemical workstation with wires. The piezoelectric voltage output was measured in 5, 15, and 30 s NIR irradiation cycle.

### Cell culture

Rat bone marrow mesenchymal stem cells (BMSCs) were isolated form the femurs and tibias of 4-week-old male Sprague–Dawley (SD) rats. The rats were sacrificed via cervical dislocation and soaked in 75% ethanol for 10 min. The tibias and femurs were separated and the epiphysis were cut under aseptic conditions. Bone marrow cavity was rinsed with minimum essential medium α (α-MEM; HyClone, USA) using 5 mL syringe to obtain bone marrow cell suspension. The flushing fluid was filtered through a 63 μm sterile cell strainer and centrifuged at 1000 rpm for 10 min to obtain the cell pellet. The cells were resuspended in α-MEM supplementing 10% fetal bovine serum (FBS) (Gibco, USA) and 1% penicillin-streptomycin (HyClone, USA) and placed in a T-25 cell culture flask, which was incubated in a humidified incubator containing 5% CO_2_ at 37 °C. The medium was changed the next day and once three days thereafter. The BMSCs were passaged every 7 days and the cells at passage 3–5 were applied in the subsequent experiments.

To evaluate the osteogenic differentiation of BMSCs, cells were cultured on the surface of the nanocomposite membranes immersed by mesenchymal stem cell osteogenic differentiation medium (α-MEM supplemented with 10% FBS, 1% penicillin-streptomycin, 10 mmol/L β-glycerophosphate (Sigma, USA), 10 nmol/L dexamethasone, and 50 µg/mL ascorbic acid).

### Cell counting Kit-8 (CCK-8) assay

Cell viability of BMSCs with nanocomposite membranes was assessed by CCK-8 assay. CCK-8 assay was implemented using Cell Counting Kit-8 (CCK-8) (Beyotime, China) according to the manufacturer’s instructions. Briefly, BMSCs were seeded onto the nanocomposite membranes in a 96-well plate at a density of 1 × 10^5^ cells per well. After seeding for 1, 3, and 5 days, the medium was replaced with culture medium containing 10% CCK-8 solution, followed by incubation for 1 h at 37 °C. The supernatant each well was then transferred into another 96-well plate with 3 replicates per group, and then the absorbance was measured at 450 nm wavelength using a microplate reader (EnSpire, PerkinElmer).

### Live/dead staining assay

Cell viability of BMSCs with nanocomposite membranes was also assessed by live/dead staining. For live/dead staining, cells were planted onto the nanocomposite membranes in a 12-well plate. After incubating for 5 days, cells were stained by Calcein-AM/PI Double Stain Kit (YEASEN, China). In brief, 200 µL staining buffer containing 2 µmol/mL calcein–AM and 1.5 µmol/mL PI was added per well and cells were incubated at 37 °C for 15 min. The fluorescent photos were captured with a confocal microscope (Nikon A1-Si).

### Alkaline phosphatase (ALP) assay

Alkaline Phosphatase Assay was measured by H5N1Alkaline Phosphatase Assay Kit (Beyotime, China) following the manufacturer’s instructions. After cultured in medium on the nanocomposite membrane for osteogenic differentiation for 3 and 7 days, the cells were harvested and lysed in RIPA buffer (P0013B, Beyotime, China). Subsequently, ALP activity of cells was detected with chromogenic substrate solution following its instructions. DEA enzyme activity units are alkaline phosphatase activity units. It is defined as the amount of alkaline phosphatase required to hydrolyze para-nitrophenyl phosphate chromogenic substrate to yield 1 µmol of p-nitrophenol per minute in diethanolamine (DEA) buffer at pH 9.8 at 37 °C is defined as a unit of enzyme activity, and is also referred to as a DEA enzyme activity unit. The absorbance was measured at 405 nm wavelength using a microplate reader (EnSpire, PerkinElmer), and the DEA enzyme activity intensity was calculated.

### Quantitative real-time PCR (qRT-PCR)

Total RNA was extracted using TRIZOL (TaKaRa, Japan) according to the manufacturer’s instructions. Complementary DNA (cDNA) was reverse transcribed from the RNA templates using HiScript III RT SuperMix for qPCR (+ gDNA wiper) (Vazyme, China). qRT-PCR was performed using ChamQ Universal SYBR qPCR Master Mix (Vazyme, China) on Step One Plus real-time PCR systems (Applied Biosystems, Thermo Fisher, USA). Quantification of target gene expressions was analyzed by the 2^−ΔΔCt^ method, normalized to the expression of glyceraldehyde 3-phosphate dehydrogenase (GAPDH) gene, and was presented as mean ± SD of replicates. The primer sequences used in qRT-PCR are described in Table S2.

### Immunofluorescence for cell staining

Cells cultured on the nanocomposite membranes were fixed with 4% paraformaldehyde for 15 min. After washed three times with PBS, 0.5% Triton X-100 was added to permeabilize the cells for 15 min. Subsequently, cells were blocked with 5% bovine serum albumin (BSA) for 1 h at room temperature, followed by incubation with primary antibodies (1:200) respectively in a humidified chamber at 4 °C overnight. Then the samples were washed three times and incubated with Coralite488-conjugated Goat Anti-Rabbit IgG(H + L) secondary antibody (1:200) (Proteintech, USA) respectively for 1 h at room temperature. Cytoskeleton was stained with TRITC Phalloidin (YEASEN, China) for 30 min and cell nuclei were counterstained with 4′,6-Diamidino-2-phenylin- dole (DAPI, Sigma, USA) for 5 min. Images were captured using a laser-scanning confocal microscope (Nikon A1-Si, Japan). The primary antibodies used in immunofluorescence are described in Table S3.

### RNA sequencing

BMSCs were cultured on PDA@PVFT/PU nanocomposite membranes in continuous and circular NIR irradiation for 7 days. Then, cell lysates were collected using TRIZOL (TaKaRa, Japan) according to the manufacturer’s instructions. Three independent replicate samples from two groups were sent to Beijing Novogene Co, Ltd. for the global transcriptome analysis. Before transcriptome sequencing, the samples were subjected to quality-control inspection, including: (i) RNA concentration > 50ng/uL, total amount > 1.5 µg; (ii) the 260 nm/280 nm absorbance ratio was nearly within the range of 1.8–2.1, and the sample was free of macromolecular pollution; (3) the sample remains intact without degradation.

In the differential expression analysis, DESeq2 was applied to identify differentially expressed genes. Differentially expressed genes (DEGs) were chosen as p-value < 0.05 and |FoldChange| ≥ 1. For Gene Ontology (GO) enrichment analysis of DEGs, clusterProfiler R package was implemented. GO terms with corrected p-value < 0.05 were considered significantly enriched by DEGs. The Kyoto Encyclopedia of Genes and Genomes (KEGG) enrichment analysis of differentially-expressed genes was also performed by clusterProfiler R package. Differentially-expressed genes were significantly enriched for KEGG pathways with a p-value less than 0.05.

### Intracellular Ca^2+^ concentration detection

Intracellular Ca^2+^ concentration was assessed by Fluo-4 AM (Beyotime, China) following the manufacturer’s protocols. BMSCs were seeded on the membranes and cultured for 24 h. After washed with phosphate-buffered saline (PBS) three times, the cells were stained by Fluo-4 AM solution (5µmol/L) for 20 min at 37 °C. The images were captured with a confocal microscope (Nikon A1-Si).

### Mitochondrial membrane potential assay

Mitochondrial membrane potential was detected by Mitochondrial Membrane Potential Assay Kit (Beyotime, China) following the manufacturer’s protocols. JC-1 is a cationic dye that exhibits potential-dependent accumulation in mitochondria. In cells with higher mitochondrial membrane potential, JC-1 aggregates in the matrix of mitochondria and forms the red fluorescent aggregates. However, in cells with lower mitochondrial membrane potential, JC-1 does not aggregate and exists as a green fluorescent monomer. After washed with JC-1 Staining Buffer three times, the cells were stained by JC-1 working solution for 30 min at 37 °C. The images were captured with a confocal microscope (Nikon A1-Si), and mitochondrial membrane potential change can be indicated by the red/green fluorescence intensity ratio.

### Animal experiments

All animal experiments were authorized by the Animal Ethics Committee of Tongji Medical College (Wuhan, China) and approved by the Institutional Animal Care and Use Committee of Tongji Medical College (Wuhan, China).

Adult male Sprague-Dawley (SD) rats at the age of 6–8 weeks were used in this study to created cranial bone defects. The SD rats were allowed to accommodate to our laboratory environment for one week before the experiment. The rats were anesthetized and surgically exposed with dorsal cranium. Then a 5-mm diameter trephine was used to create bilateral cranial defect models. Two parallel circular full-thickness bone defects were cut out of the skull and covered with different membranes. The treatments were divided into six groups (*n* ≥ 6 per group): (1) sutured without membrane (Control), (2) covered with PVFT membrane (PVFT), (3) covered with PVFT/PU nanocomposite membrane (PVFT/PU), (4) covered with PDA@PVFT/PU nanocomposite membrane without NIR irradiation [PDA@PVFT/PU(NIR-)], (5) covered with PDA@PVFT/PU nanocomposite membrane with continuous NIR irradiation [PDA@PVFT/PU(NIR+, Con)], (6) covered with PDA@PVFT/PU nanocomposite membrane with circular NIR irradiation [PDA@PVFT/PU(NIR+, Cir)]. After suture removed, Rats implanted PDA@PVFT/PU nanocomposite membranes started to be treated with continuous or circular NIR irradiation (0.5 W/cm^2^, 10 min) every day on the right-side defects, with the left side untreated. For 30 days and 60 days after treatment, the rats were sacrificed respectively to acquire the skull specimens for further evaluation.

### Micro-CT scanning evaluation

The cranial specimens were fixed with 4% paraformaldehyde for 24 h. Then the samples were scanned using a micro-CT scanner (Inveon Multimodality Scanner; Siemens, Erlangen, Germany) at a resolution of 9 μm to assess new bone formation. After 3D reconstruction of the images by the micro-CT system software package, the region of interest (ROI) was circled around the defect area at a diameter of 5 mm. Then, the percent bone volume ratio (bone volume/tissue volume, BV/TV), bone surface density (bone surface/tissue volume, BS/TV) and trabecular numbers (Trabecular Number, Tb. N) in the reconstructed ROI were calculated analyzed evaluate the level of bone regeneration.

### Hematoxylin Eosin (H&E) staining

The skull specimens were decalcified with 10% EDTA decalcification solution after micro-CT scanning. When the bone tissue became soft, the samples were processed according to routine Hematoxylin Eosin (H&E) Staining procedures for morphological observation. Briefly, the samples were dehydrated, embedded in paraffin and cut into 4 μm thick sections. After deparaffinization and rehydration, the sections were subjected to hematoxylin staining and differentiated with acid alcohol. Subsequently, the specimen sections were stained with eosin solution, followed by dehydration and clearing. Histological images were captured by an optical microscope.

### Masson’s trichrome staining

The paraffin sections were prepared as described above. For Masson’s trichrome staining, the tissue sections were first immersed in hematoxylin solution for nuclei staining. After differentiation, the sections were stained with trichrome staining solution, followed by routine dehydration and clearing procedure. Relative images were obtained using an optical microscope.

### Immunofluorescence for tissue staining

The paraffin-embedded cranial tissue sections were blocked with 5% bovine serum albumin (BSA) for 1 h to avoid non-specific staining. Subsequently, the sections were incubated with primary antibodies COL1A1 (1:200, ABclonal, China, A1352) and RUNX2 (1:200, ABclonal, China, A2851) at 4 ℃ overnight in a lucifugal chamber. The slides were then washed with PBS for three times, followed by incubation with Coralite488-conjugated Goat Anti-Rabbit IgG(H + L) secondary antibody (1:200) (Proteintech, USA) for 1 h in the dark. Cell nuclei were stained with DAPI for 5 min. The fluorescent images were captured with a confocal microscope (Nikon A1-Si, Japan). The primary antibodies used in immunofluorescence are described in Table S2.

### Immunohistochemistry staining

The paraffin sections were prepared as described previously. The tissue sections were placed in EDTA antigen retrieval buffer (pH 9.0) and subjected to antigen retrieval in a microwave oven. After cooling, the slides were washed three times in PBS for 5 min each. Subsequently, the sections were incubated in 3% hydrogen peroxide solution at room temperature in the dark for 25 min to inhibit endogenous peroxidase activity. Then, 5% BSA was applied to the tissue sections to block non-specific binding at room temperature for 30 min. Afterwards, the sections were incubated overnight with relevant primary antibodies OPN (1:200, ABclonal, China, A1499) at 4 °C. After washing, the tissue sections were incubated with HRP-conjugated secondary antibodies (K5007, DAKO, Germany) corresponding to the species of the primary antibodies at room temperature for 1 h. After washing, fresh DAB coloration solution (K5007, DAKO, Germany) was added, and the reaction was stopped by rinsing the sections with distilled water. The sections were then counterstained with Harris hematoxylin for approximately 3 min, differentiated in 1% hydrochloric acid alcohol for a few seconds, and blued with 1% ammonia water before being rinsed with running water. Finally, the sections were dehydrated and cleared in 75% and 85% ethanol, absolute ethanol, and xylene, and then transferred from xylene to neutral balsam for mounting. Images were obtained using an optical microscope.

### Statistical analysis

All numerical data were presented as mean ± standard deviation (SD) and statistically analyzed using GraphPad Prism 8.0 (GraphPad Software Inc., USA) with three or more replicate values. Significant differences were determined using Student’s t-test for comparison of two groups and one-way ANOVA with post-hoc Tukey’s test for multiple comparisons. The statistical significance was declared when *p < 0.05, **p < 0.01, ***p < 0.001 and ****p < 0.0001.

## Conclusion

In conclusion, we successfully developed an electrospun membrane containing piezoelectric fibers inside, which was incorporated with HEPCP and equipped by photothermal-sensitive agents. The flexible piezoelectric membrane was applied for bone regeneration which can respond to a remote NIR irradiation to achieve the biomimetic mechano-electrical coupling. Besides, cyclic NIR stimulation was applied to simulate the natural rule of diurnal shift of mechano-electrical coupling, which predominantly contributes to enhanced osteogenic differentiation of BMSCs. This could mainly be explained by promotion in the cellular sensing and transduction of mechanical signals. Our study thus provides a feasible and high-performance strategy combining efficient electrical and mechanical cues with local NIR application for promotion of bone repair, highlighting the promise of mechano-electrical coupling for clinical use in tissue repair.

### Electronic supplementary material

Below is the link to the electronic supplementary material.

Supplementary Material 1

## Data Availability

All data can be found in the manuscript or in the Supplementary Material. The data and material of this study is available from the corresponding authors on reasonable request.

## References

[1] Zhou X, Sun J, Wo K, Wei H, Lei H, Zhang J, Lu X, Mei F, Tang Q, Wang Y, Luo Z, Fan L, Chu Y, Chen L (2022). nHA-Loaded Gelatin/Alginate hydrogel with combined physical and bioactive features for maxillofacial bone repair. Carbohydr Polym.

[2] Xiong Y, Lin Z, Bu P, Yu T, Endo Y, Zhou W, Sun Y, Cao F, Dai G, Hu Y, Lu L, Chen L, Cheng P, Zha K, Shahbazi M-A, Feng Q, Mi B, Liu GA. Whole-course-repair system based on neurogenesis-angiogenesis crosstalk and macrophage reprogramming promotes Diabetic Wound Healing. Adv Mater 2023, 35 (19), e2212300. 10.1002/adma.202212300 36811203

[3] Gi B, Sp B, Yw S, Jk C, Chf H, Uw J, Re J. Randomized controlled clinical trial comparing guided bone regeneration of Peri-implant defects with soft-type Block versus Particulate Bone substitutes: six-Month results of hard-tissue changes. J Clin Periodontol 2022, *49* (5). 10.1111/jcpe.13606 35191065

[4] Hr M, E E, Djr G, Bes A, Lg F, Pa P, Jm deA. Association of Hyaluronic Acid with a deproteinized bovine graft improves bone repair and increases bone formation in critical-size bone defects. J Periodontol 2021, *92* (11). 10.1002/JPER.20-0613 33258112

[5] Tikhonova AN, Dolgalev I, Hu H, Sivaraj KK, Hoxha E, Cuesta-Domínguez Á, Pinho S, Akhmetzyanova I, Gao J, Witkowski M, Guillamot M, Gutkin MC, Zhang Y, Marier C, Diefenbach C, Kousteni S, Heguy A, Zhong H, Fooksman DR, Butler JM, Economides A, Frenette PS, Adams RH, Satija R, Tsirigos A (2019). Aifantis, I. The bone marrow microenvironment at single-cell resolution. Nature.

[6] Cai X, Wang K-C, Meng Z. Mechanoregulation of YAP and TAZ in Cellular Homeostasis and Disease Progression. *Frontiers in Cell and Developmental Biology* 2021, *9*. 10.3389/fcell.2021.673599 PMC8182050 34109179

[7] Gvaramia D, Müller E, Müller K, Atallah P, Tsurkan M, Freudenberg U, Bornhäuser M, Werner C (2017). Combined influence of Biophysical and biochemical cues on maintenance and proliferation of hematopoietic stem cells. Biomaterials.

[8] Benoit DSW, Schwartz MP, Durney AR, Anseth KS (2008). Small functional groups for controlled differentiation of hydrogel-encapsulated human mesenchymal stem cells. Nat Mater.

[9] Vining KH, Mooney DJ (2017). Mechanical forces direct stem cell Behaviour in Development and Regeneration. Nat Rev Mol Cell Biol.

[10] Sun J, Zhang J, Yang L, Zhang C, Wang Y, Lei H, Wo K, Fan W, Zhao B, Wang J, Shi Y, Luo Z, Su B, Song J, Chu Y, Chen L (2024). Piezocatalytic Strategy facilitates Diabetic Bone regeneration through high-performance anti-oxidative recycling. Chem Eng J.

[11] Li W, Yan Z, Ren J, Qu X (2018). Manipulating cell fate: dynamic control of cell behaviors on functional platforms. Chem Soc Rev.

[12] Li Y, Xiao Y, Liu C (2017). The Horizon of Materiobiology: a perspective on material-guided cell behaviors and tissue Engineering. Chem Rev.

[13] Tu C, Xiao Y, Ma Y, Wu H, Song M (2018). The Legacy effects of Electromagnetic fields on Bone Marrow Mesenchymal Stem Cell Self-Renewal and multiple differentiation potential. Stem Cell Res Ther.

[14] Chen L, Qu J, Xiang C (2019). The multi-functional roles of menstrual blood-derived stem cells in Regenerative Medicine. Stem Cell Res Ther.

[15] Seong Y, Moon J, Kim J (2014). Egr1 mediated the neuronal differentiation Induced by extremely low-frequency Electromagnetic fields. Life Sci.

[16] Liu J, Zeng H, Xiao P, Yang A, Situ X, Wang Y, Zhang X, Li W, Pan W, Wang Y (2020). Sustained release of Magnesium Ions mediated by a dynamic mechanical hydrogel to enhance BMSC proliferation and differentiation. ACS Omega.

[17] Kim K, Dean D, Wallace J, Breithaupt R, Mikos AG, Fisher JP (2011). The influence of Stereolithographic Scaffold Architecture and Composition on Osteogenic Signal Expression with rat bone marrow stromal cells. Biomaterials.

[18] Zhang X, Zhang C, Lin Y, Hu P, Shen Y, Wang K, Meng S, Chai Y, Dai X, Liu X, Liu Y, Mo X, Cao C, Li S, Deng X, Chen L (2016). Nanocomposite membranes enhance bone regeneration through restoring physiological Electric Microenvironment. ACS Nano.

[19] Bai Y, Zheng X, Zhong X, Cui Q, Zhang S, Wen X, Heng BC, He S, Shen Y, Zhang J, Wei Y, Deng X, Zhang X. Manipulation of heterogeneous Surface Electric potential promotes Osteogenesis by strengthening RGD peptide binding and Cellular Mechanosensing. Adv Mater 2023, 35 (24), e2209769. 10.1002/adma.202209769 36934418

[20] Liu Y, Dzidotor G, Le TT, Vinikoor T, Morgan K, Curry EJ, Das R, McClinton A, Eisenberg E, Apuzzo LN, Tran KTM, Prasad P, Flanagan TJ, Lee S-W, Kan H-M, Chorsi MT, Lo KWH, Laurencin CT, Nguyen TD (2022). Exercise-Induced Piezoelectric Stimulation for cartilage regeneration in rabbits. Sci Transl Med.

[21] Jiang Y, Trotsyuk AA, Niu S, Henn D, Chen K, Shih C-C, Larson MR, Mermin-Bunnell AM, Mittal S, Lai J-C, Saberi A, Beard E, Jing S, Zhong D, Steele SR, Sun K, Jain T, Zhao E, Neimeth CR, Viana WG, Tang J, Sivaraj D, Padmanabhan J, Rodrigues M, Perrault DP, Chattopadhyay A, Maan ZN, Leeolou MC, Bonham CA, Kwon SH, Kussie HC, Fischer KS, Gurusankar G, Liang K, Zhang K, Nag R, Snyder MP, Januszyk M, Gurtner GC, Bao Z (2023). Wireless, Closed-Loop, Smart Bandage with Integrated Sensors and stimulators for Advanced Wound Care and Accelerated Healing. Nat Biotechnol.

[22] da Silva RA, Xue R, de Torresi SIC, Cartmell S (2022). Capacitive Electrical Stimulation of a conducting Polymeric Thin Film induces human mesenchymal stem cell osteogenesis. Biointerphases.

[23] Srirussamee K, Xue R, Mobini S, Cassidy NJ, Cartmell SH (2021). Changes in the Extracellular Microenvironment and osteogenic responses of mesenchymal Stem/Stromal cells Induced by in Vitro Direct Electrical Stimulation. J Tissue Eng.

[24] Rotherham M, Nahar T, Broomhall TJ, Telling ND, El Haj AJ (2022). Remote magnetic actuation of cell signalling for tissue Engineering. Curr Opin Biomedical Eng.

[25] Santos LJ, Reis RL, Gomes ME (2015). Harnessing magnetic-mechano actuation in Regenerative Medicine and tissue Engineering. Trends Biotechnol.

[26] Di X, Gao X, Peng L, Ai J, Jin X, Qi S, Li H, Wang K, Luo D (2023). Cellular Mechanotransduction in Health and diseases: from molecular mechanism to therapeutic targets. Signal Transduct Target Ther.

[27] Park HW, Kim YC, Yu B, Moroishi T, Mo J-S, Plouffe SW, Meng Z, Lin KC, Yu F-X, Alexander CM, Wang C-Y, Guan K-L (2015). Alternative wnt signaling activates YAP/TAZ. Cell.

[28] Li X, Serdijn WA, Zheng W, Tian Y, Zhang B (2015). The Injectable Neurostimulator: an emerging therapeutic device. Trends Biotechnol.

[29] Cook JJ, Summers NJ, Cook EA (2015). Healing in the New Millennium: bone stimulators: an overview of where we’ve been and where we may be heading. Clin Podiatr Med Surg.

[30] Pohling C, Nguyen H, Chang E, Schubert KE, Nie Y, Bashkirov V, Yamamoto V, Zeng Y, Stupp R, Schulte RW, Patel CB (2023). Current status of the Preclinical Evaluation of Alternating Electric Fields as a form of Cancer Therapy. Bioelectrochemistry.

[31] Özmen T, Koparal SS, Karataş Ö, Eser F, Özkurt B, Gafuroğlu TÜ (2021). Comparison of the clinical and Sonographic effects of Ultrasound Therapy, extracorporeal shock Wave Therapy, and Kinesio Taping in lateral epicondylitis. Turk J Med Sci.

[32] Jin R, Xu J, Duan L, Gao G (2021). Chitosan-Driven skin-attachable hydrogel sensors toward human motion and physiological Signal Monitoring. Carbohydr Polym.

[33] Woeppel KM, Cui XT. Nanoparticle and Biomolecule Surface Modification synergistically increases neural Electrode Recording Yield and minimizes inflammatory host response. Adv Healthc Mater 2021, 10 (16), e2002150. 10.1002/adhm.202002150 PMC8373793 34190425

[34] Krylov VV, Osipova EA, Pankova NA, Talikina MG, C Yu. V., I Yu. G., B A. A., N V. A. The Effect of a temporal shift in diurnal geomagnetic variation on Roach Rutilus Rutilus L. Embryos: a comparison with effects of simulated geomagnetic storms. BIOPHYSICS. 2017;62(4):675–81.

[35] Huang X, Xing J, Wang Z, Han J, Wang R, Li C, Xiao C, Lu F, Zhai J, Zhou Z, Li Y, Zhou L, Song Z, Chen D, Yu P, Ning C, Jiang X (2021). 0D/1D Heterojunction Implant with Electro-Mechanobiological Coupling cues promotes Osteogenesis. Adv Funct Mater.

[36] Kamkin A, Kiseleva I, Isenberg G, Wagner KD, Günther J, Theres H, Scholz H (2003). Cardiac fibroblasts and the Mechano-Electric Feedback mechanism in healthy and diseased hearts. Prog Biophys Mol Biol.

[37] Liu Z, Cai M, Zhang X, Yu X, Wang S, Wan X, Wang ZL, Li L. Cell-traction-triggered On-Demand Electrical Stimulation for Neuron-Like differentiation. Adv Mater 2021, 33 (51), e2106317. 10.1002/adma.202106317 34655105

[38] Ferreira SA, Motwani MS, Faull PA, Seymour AJ, Yu TTL, Enayati M, Taheem DK, Salzlechner C, Haghighi T, Kania EM, Oommen OP, Ahmed T, Loaiza S, Parzych K, Dazzi F, Varghese OP, Festy F, Grigoriadis AE, Auner HW, Snijders AP, Bozec L, Gentleman E (2018). Bi-directional cell-pericellular matrix interactions direct stem cell fate. Nat Commun.

[39] Baker BM, Trappmann B, Wang WY, Sakar MS, Kim IL, Shenoy VB, Burdick JA, Chen CS (2015). Cell-mediated Fibre Recruitment drives Extracellular Matrix Mechanosensing in Engineered Fibrillar Microenvironments. Nat Mater.

[40] Pi Z, Zhang J, Wen C, Zhang Z, Wu D (2014). Flexible Piezoelectric Nanogenerator made of Poly(Vinylidenefluoride-Co-Trifluoroethylene) (PVDF-TrFE) thin Film. Nano Energy.

[41] Ghafari E, Jiang X, Lu N (2018). Surface morphology and Beta-phase formation of single polyvinylidene fluoride (PVDF) composite nanofibers. Adv Compos Hybrid Mater.

[42] Adeli B, Gharehaghaji AA, Jeddi AAA (2021). A feasibility study on production and optimization of PVDF/PU Polyblend Nanofiber Layers using Expert Design Analysis. Iran Polym J.

[43] Li Y, Fu R, Duan Z, Zhu C, Fan D (2022). Artificial Nonenzymatic antioxidant MXene Nanosheet-Anchored Injectable Hydrogel as a mild photothermal-controlled oxygen release platform for Diabetic Wound Healing. ACS Nano.

[44] Sun X, Li L, Zhang H, Dong M, Wang J, Jia P, Bu T, Wang X, Wang L. Near-Infrared light-regulated drug-food homologous bioactive molecules and Photothermal Collaborative Precise Antibacterial Therapy Nanoplatform with Controlled Release Property. Adv Healthc Mater 2021, 10 (16), e2100546. 10.1002/adhm.202100546 34081401

[45] Gao Y, Geng X, Wang X, Han N, Zhang X, Li W (2021). Synthesis and characterization of Microencapsulated Phase Change materials with Chitosan-based polyurethane Shell. Carbohydr Polym.

[46] Gama NV, Amaral C, Silva T, Vicente R, Coutinho JAP, Barros-Timmons A, Ferreira A (2018). Thermal Energy Storage and mechanical performance of crude glycerol polyurethane composite foams containing phase change materials and expandable Graphite. Mater (Basel).

[47] Niu Z, Yuan W (2021). Smart Nanocomposite Nonwoven Wearable Fabrics Embedding Phase Change materials for highly efficient Energy Conversion–Storage and Use as a stretchable conductor. ACS Appl Mater Interfaces.

[48] Li M, Wang Y, Yu Z, Fu Y, Zheng J, Liu Y, Cui J, Zhou H, Li D (2020). Self-powered infrared-responsive electronic skin employing Piezoelectric Nanofiber nanocomposites Driven by Microphase Transition. ACS Appl Mater Interfaces.

[49] Li B, Liu F, Ye J, Cai X, Qian R, Zhang K, Zheng Y, Wu S, Han Y. Regulation of macrophage polarization through periodic photo-thermal treatment to facilitate Osteogenesis. Small 2022, 18 (38), e2202691. 10.1002/smll.202202691 35986434

[50] Tong L, Liao Q, Zhao Y, Huang H, Gao A, Zhang W, Gao X, Wei W, Guan M, Chu PK, Wang H (2019). Near-Infrared Light Control of Bone Regeneration with Biodegradable Photothermal Osteoimplant. Biomaterials.

[51] Wu M, Liu H, Zhu Y, Chen F, Chen Z, Guo L, Wu P, Li G, Zhang C, Wei R, Cai L (2023). Mild photothermal-stimulation based on Injectable and Photocurable Hydrogels orchestrates Immunomodulation and Osteogenesis for high-performance bone regeneration. Small.

[52] Wu Y, Liao Q, Wu L, Luo Y, Zhang W, Guan M, Pan H, Tong L, Chu PK, Wang H (2021). ZnL2-BPs Integrated Bone Scaffold under Sequential Photothermal Mediation: a Win-Win Strategy delivering Antibacterial Therapy and fostering Osteogenesis thereafter. ACS Nano.

[53] Dey Bhowmik A, Das T, Chattopadhyay A (2023). Chronic exposure to environmentally relevant concentration of Fluoride impairs osteoblast’s collagen synthesis and matrix mineralization: involvement of epigenetic regulation in skeletal fluorosis. Environ Res.

[54] Garibaldi N, Contento BM, Babini G, Morini J, Siciliani S, Biggiogera M, Raspanti M, Marini JC, Rossi A, Forlino A, Besio R (2021). Targeting Cellular stress in Vitro improves osteoblast homeostasis, Matrix Collagen Content and Mineralization in two murine models of Osteogenesis Imperfecta. Matrix Biol.

[55] Desbois C, Karsenty G (1995). Osteocalcin cluster: implications for functional studies. J Cell Biochem.

[56] Hosseini S, Naderi-Manesh H, Vali H, Baghaban Eslaminejad M, Azam Sayahpour F, Sheibani S, Faghihi S (2019). Contribution of osteocalcin-mimetic peptide enhances osteogenic activity and Extracellular Matrix mineralization of human osteoblast-like cells. Colloids Surf B Biointerfaces.

[57] Jing H, Su X, Gao B, Shuai Y, Chen J, Deng Z, Liao L, Jin Y (2018). Epigenetic inhibition of wnt pathway suppresses osteogenic differentiation of BMSCs during osteoporosis. Cell Death Dis.

[58] Zhu W, Yan K, Chen X, Zhao W, Wu Y, Tang H, Chen M, Wu J, Wang P, Zhang R, Shen Y, Zhang DA (2021). Founder pathogenic variant of PPIB Unique to Chinese Population Causes Osteogenesis Imperfecta IX. Front Genet.

[59] Wu Y, Wu J, Huang X, Zhu X, Zhi W, Wang J, Sun D, Chen X, Zhu X, Zhang X (2023). Accelerated osteogenesis of bone graft by optimizing the bone Microenvironment formed by electrical signals dependent on driving Micro Vibration Stimulation. Mater Today Bio.

[60] Zhang J, Li M, Kang E-T, Neoh KG (2016). Electrical stimulation of adipose-derived mesenchymal stem cells in Conductive scaffolds and the roles of Voltage-gated Ion channels. Acta Biomater.

[61] Perelman A, Wachtel C, Cohen M, Haupt S, Shapiro H, Tzur A. JC-1: alternative excitation wavelengths facilitate mitochondrial membrane potential cytometry. Cell Death Dis 2012, 3 (11), e430. 10.1038/cddis.2012.171 PMC3542606 23171850

[62] Pizzo P, Drago I, Filadi R, Pozzan T (2012). Mitochondrial Ca^2+^ homeostasis: mechanism, role, and tissue specificities. Pflugers Arch.

[63] Wan M-C, Tang X-Y, Li J, Gao P, Wang F, Shen M-J, Gu J-T, Tay F, Chen J-H, Niu L-N, Xiao Y-H, Jiao K (2021). Upregulation of mitochondrial dynamics is responsible for osteogenic differentiation of mesenchymal stem cells cultured on self-mineralized collagen membranes. Acta Biomater.

[64] Yu F-X, Guan K-L (2013). The Hippo pathway: regulators and regulations. Genes Dev.

[65] Elosegui-Artola A, Andreu I, Beedle AEM, Lezamiz A, Uroz M, Kosmalska AJ, Oria R, Kechagia JZ, Rico-Lastres P, Le Roux A-L, Shanahan CM, Trepat X, Navajas D, Garcia-Manyes S (2017). Roca-Cusachs, P. Force triggers YAP Nuclear Entry by regulating transport across Nuclear pores. Cell.

[66] Nardone G, Oliver-De La Cruz J, Vrbsky J, Martini C, Pribyl J, Skládal P, Pešl M, Caluori G, Pagliari S, Martino F, Maceckova Z, Hajduch M, Sanz-Garcia A, Pugno NM, Stokin GB, Forte GYAP (2017). Regulates cell mechanics by Controlling Focal Adhesion Assembly. Nat Commun.

[67] Pan H, Xie Y, Zhang Z, Li K, Hu D, Zheng X, Fan Q, Tang T (2017). YAP-Mediated mechanotransduction regulates osteogenic and adipogenic differentiation of BMSCs on hierarchical structure. Colloids Surf B.

[68] Wei Q, Holle A, Li J, Posa F, Biagioni F, Croci O, Benk AS, Young J, Noureddine F, Deng J, Zhang M, Inman GJ, Spatz JP, Campaner S, Cavalcanti-Adam EA (2020). BMP‐2 signaling and mechanotransduction synergize to drive osteogenic differentiation via YAP/TAZ. Adv Sci (Weinh).

[69] LaGuardia JS, Shariati K, Bedar M, Ren X, Moghadam S, Huang KX, Chen W, Kang Y, Yamaguchi DT, Lee JC. Convergence of Calcium Channel Regulation and mechanotransduction in skeletal regenerative Biomaterial Design. Adv Healthc Mater 2023, 12 (27), e2301081. 10.1002/adhm.202301081 PMC10615747 37380172

[70] Jin P, Jan LY, Jan Y-N (2020). Mechanosensitive Ion channels: structural features relevant to mechanotransduction mechanisms. Annu Rev Neurosci.

[71] Beech DJ, Kalli AC (2019). Force Sensing by Piezo Channels in Cardiovascular Health and Disease. Arterioscler Thromb Vasc Biol.

[72] Mammadova-Bach E, Gudermann T, Braun A (2023). Platelet mechanotransduction: Regulatory Cross Talk between Mechanosensitive receptors and Calcium channels. Arterioscler Thromb Vasc Biol.

[73] Uzieliene I, Bernotas P, Mobasheri A, Bernotiene E (2018). The role of physical stimuli on Calcium channels in Chondrogenic differentiation of mesenchymal stem cells. Int J Mol Sci.

