# Supplementary material for: Remote coupling of electrical and mechanical cues by diurnal photothermal irradiation synergistically promotes bone regeneration
Source: J Nanobiotechnology. 2024 Jul 11;22:410. doi: 10.1186/s12951-024-02671-6 (PMC11238389; doi:10.1186/s12951-024-02671-6)
Supplement: Supplementary file 1 — Supplementary Material 1 [file 12951_2024_2671_MOESM1_ESM.docx]

**Supplementary Figures and Figure Legends**


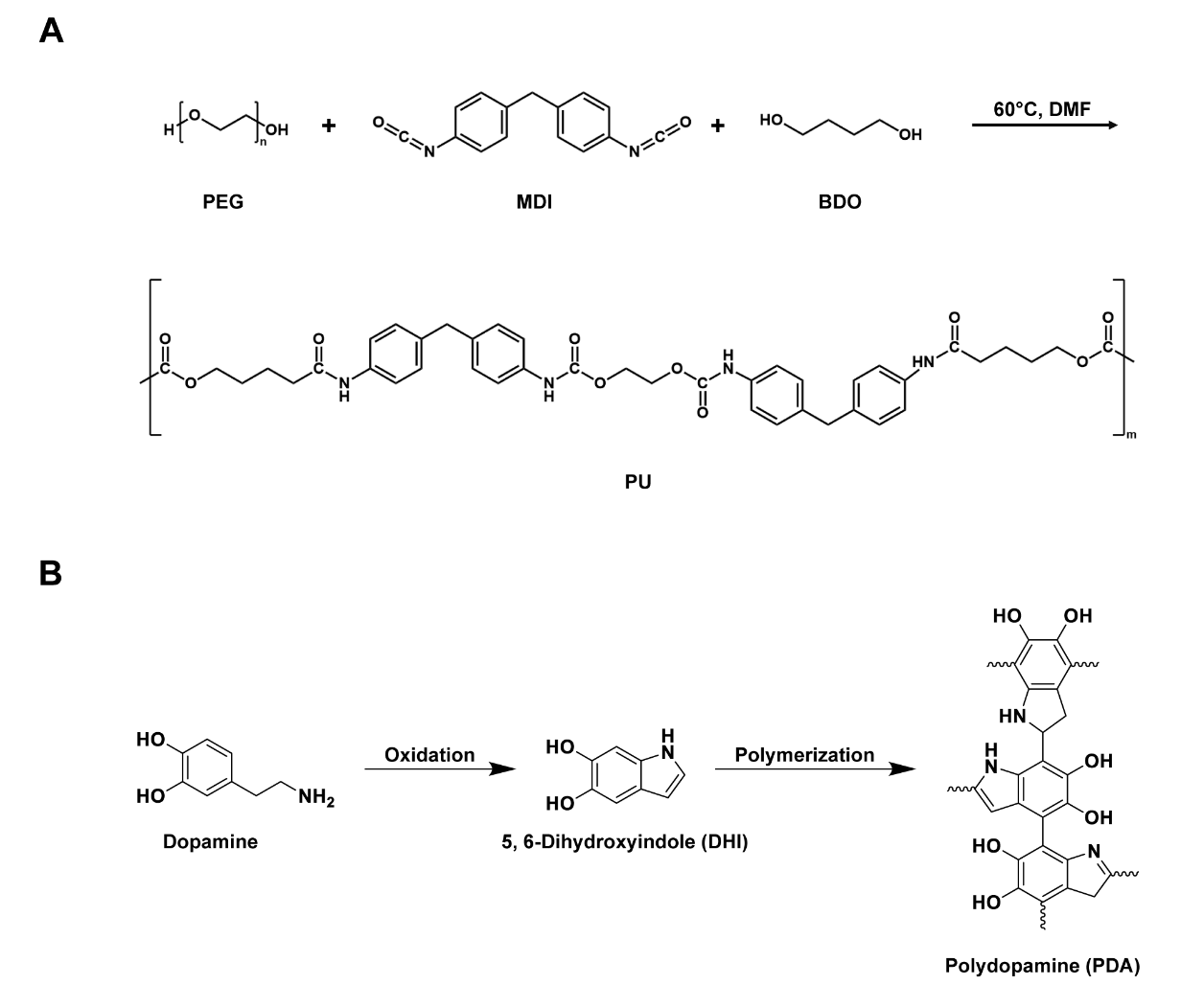


**Supplementary Figure 1.** Preparation flow of (A) PU and (B) PDA.


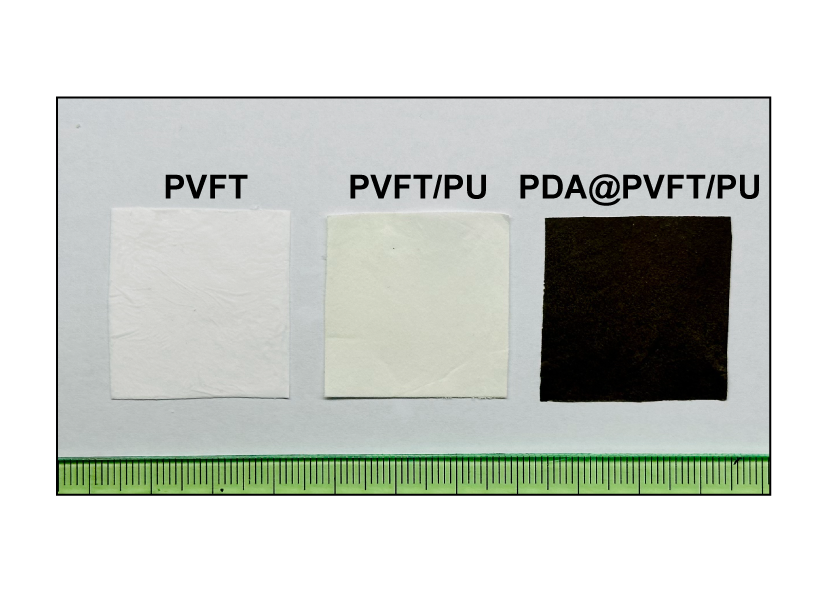


**Supplementary Figure 2.** Photograph of PVFT, PVFT/PU and PDA@PVFT/PU nanocomposite membranes.


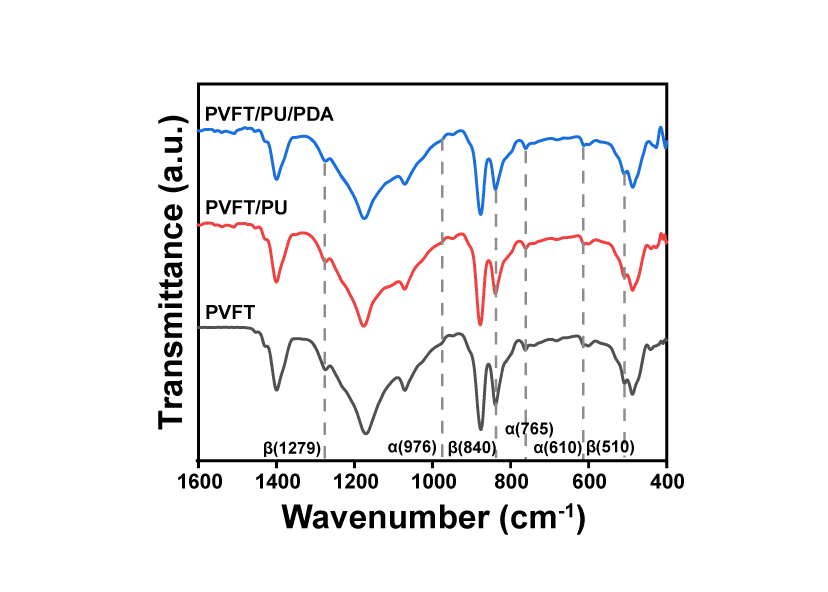


**Supplementary Figure 3.** ATR spectrum of PVFT, PVFT/PU and PDA@PVFT/PU nanocomposite membranes.


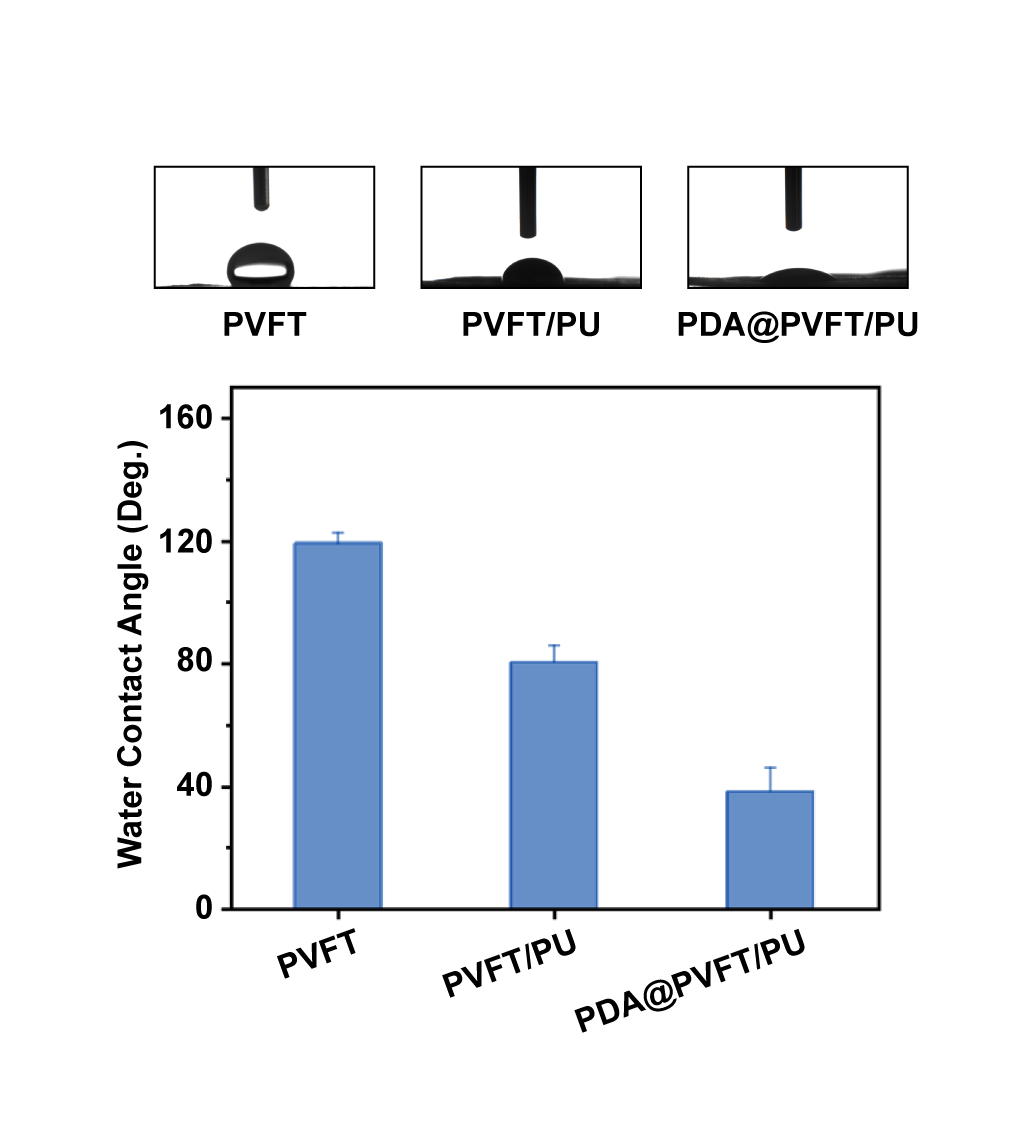


**Supplementary Figure 4.** Images and analysis of the water contact angles on the surface of PVFT, PVFT/PU and PDA@PVFT/PU nanocomposite membranes.


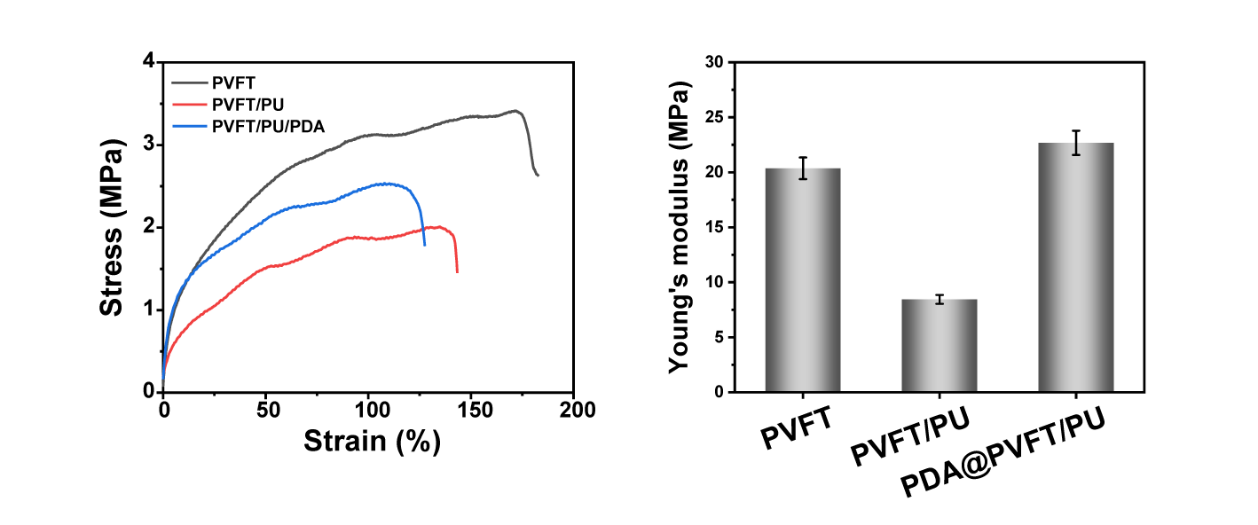


**Supplementary Figure 5.** Mechanical properties of PVFT, PVFT/PU and PDA@PVFT/PU nanocomposite membrane.


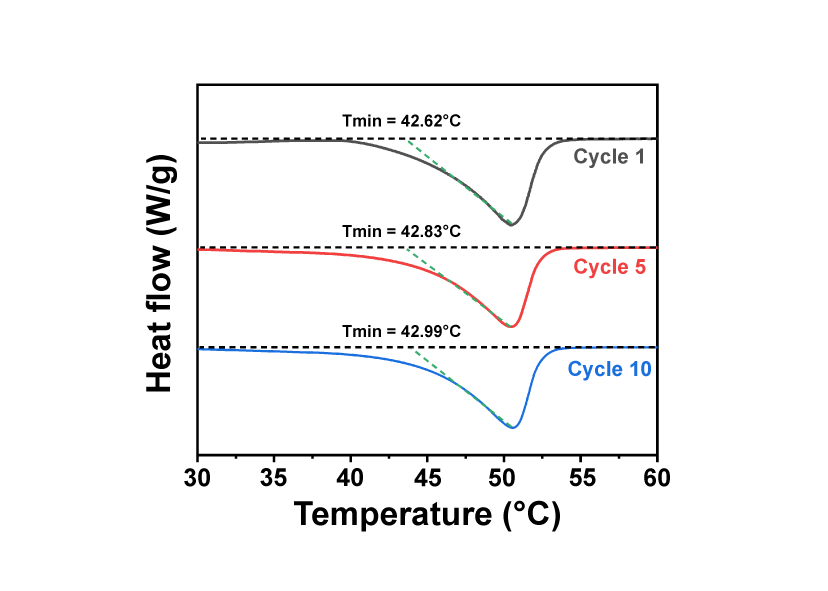


**Supplementary Figure 6.** Analysis of Minimum phase transition temperature in DSC curves of PU within 10 heating-cooling cycles.


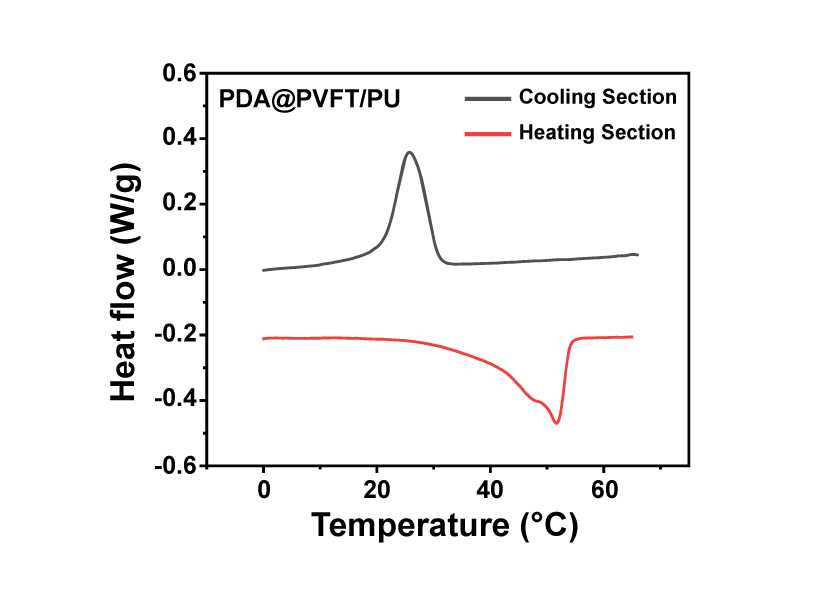


**Supplementary Figure 7.** DSC curves of PDA@PVFT/PU nanocomposite membranes during NIR irradiation.


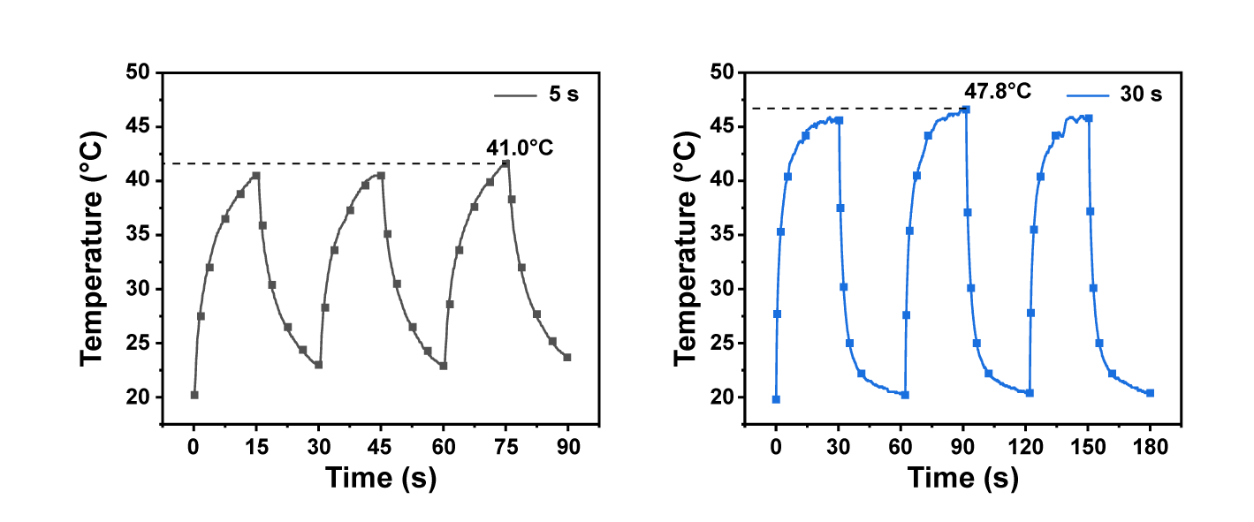


**Supplementary Figure 8.** Temperature variations of PDA@PVFT/PU nanocomposite membranes within 5s and 30s NIR irradiation cycles respectively.


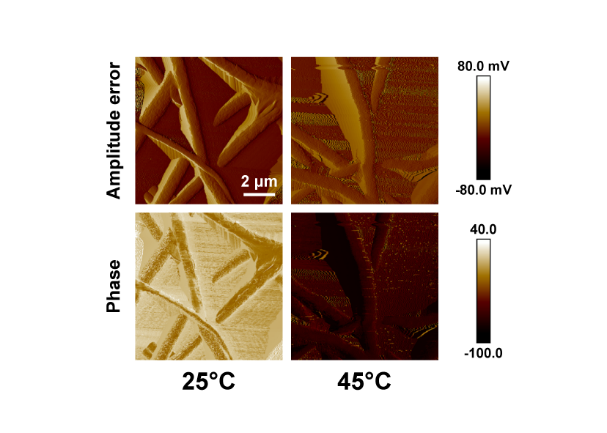


**Supplementary Figure 9.** Amplitude errors and Phase images of the PVFT NFs of PDA@PVFT/PU nanocomposite membranes under NIR irradiation cycling (Scale bar = 2 μm).


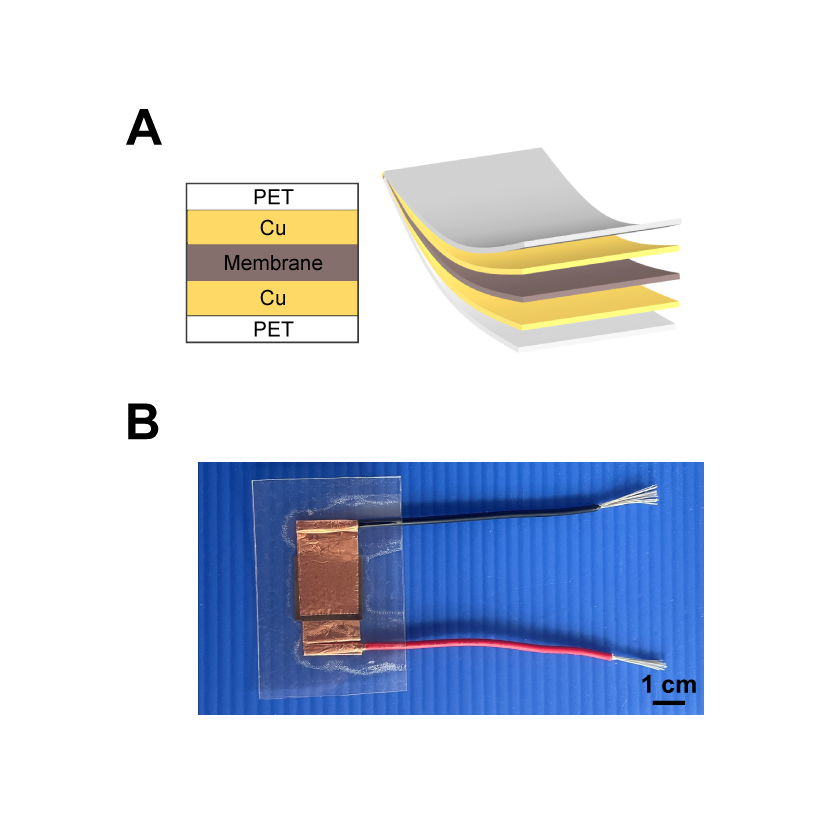


**Supplementary Figure 10.** (A) Schematic diagram and (B) experimental device for detection of piezoelectric property of nanocomposite membranes (Scale bar = 1 cm).


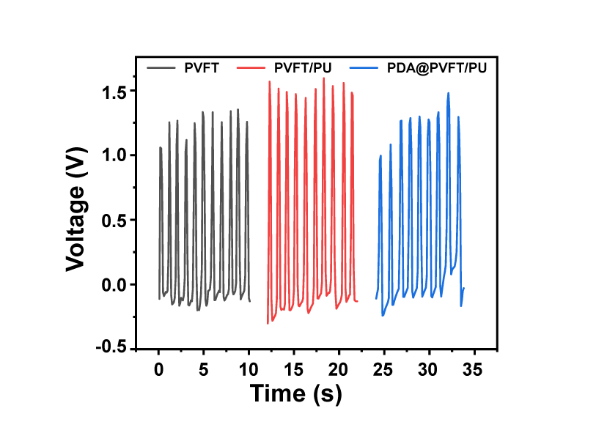


**Supplementary Figure 11.** The piezoelectric output of PVFT, PVFT/PU and PDA@PVFT/PU nanocomposite membrane induced by a constant mechanical force of 5 N, 0.5 Hz from the vertical direction.


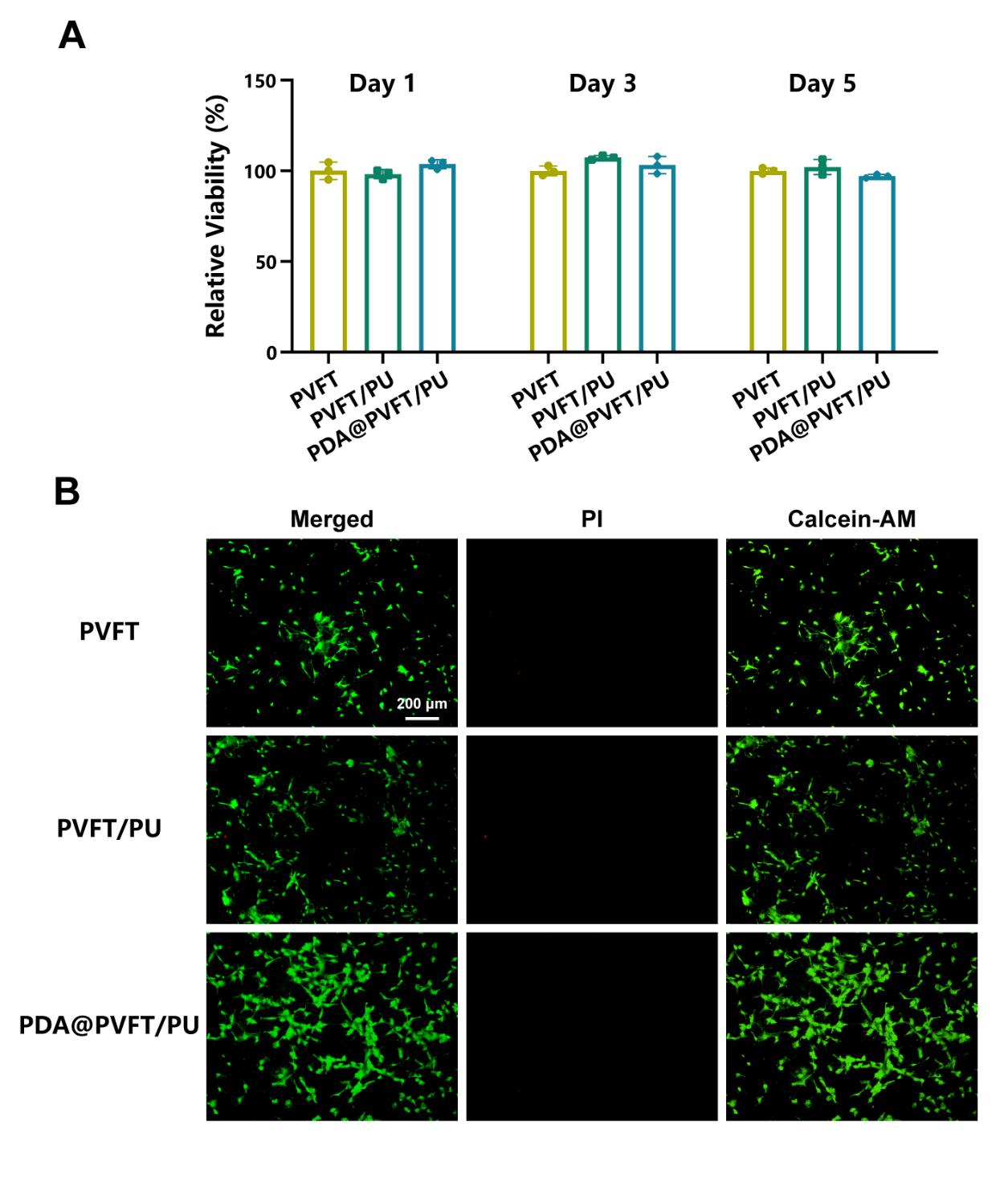


**Supplementary Figure 12. Biocompatibility of nanocomposite membranes.** (A) CCK8 assay showing the cell proliferation status of BMSCs after culturing for 1, 3, and 5 days (n = 3). (B) Fluorescence images showing live calcein-stained (green) and dead propidium iodide-labeled (red) BMSCs after culturing for 5 days (Scale bar = 200 μm).


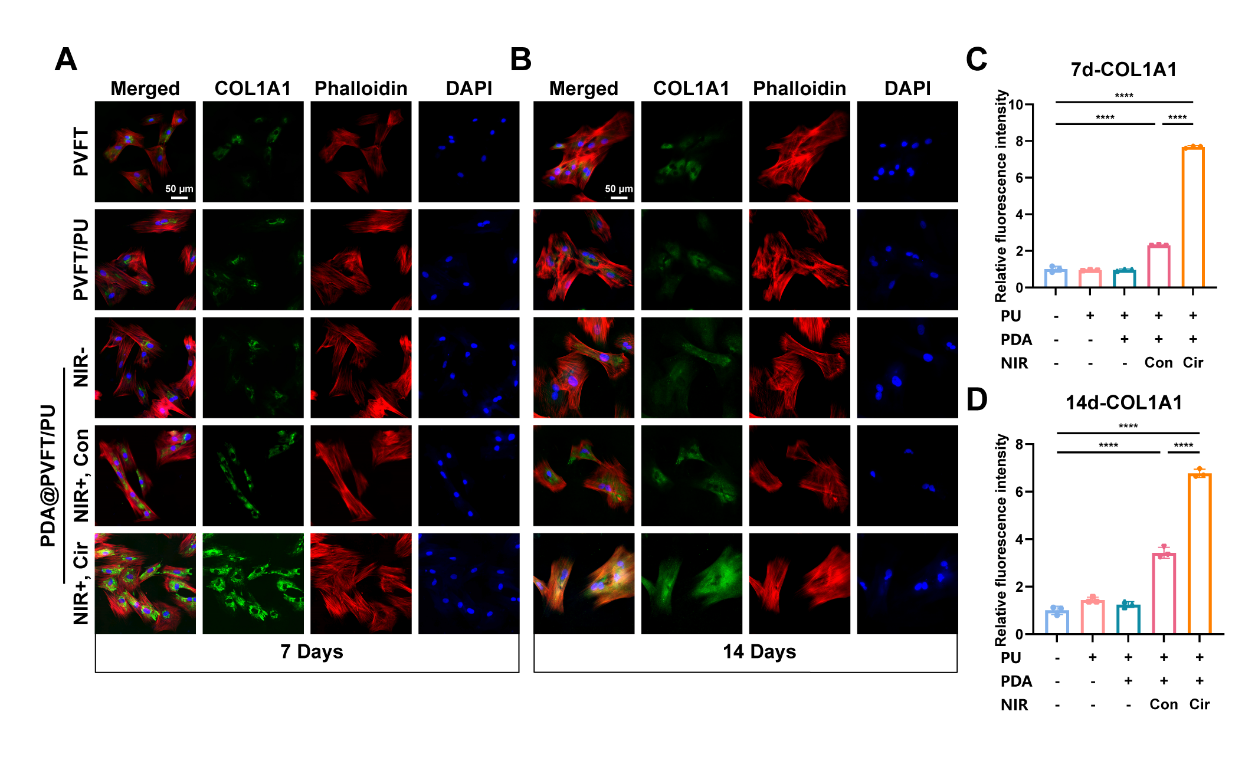

**Supplementary Figure 13.** Representative immunostaining images and quantitative analysis of COL1A1 (green), Phalloidin (red) and cell nuclei (DAPI, blue) of BMSCs cultured in osteogenic differentiation medium for 7 and 14 days (Scale bar = 50 μm). *p < 0.05, **p < 0.01, ***p < 0.001 and ****p <0.0001 (n ≥ 3 per group).


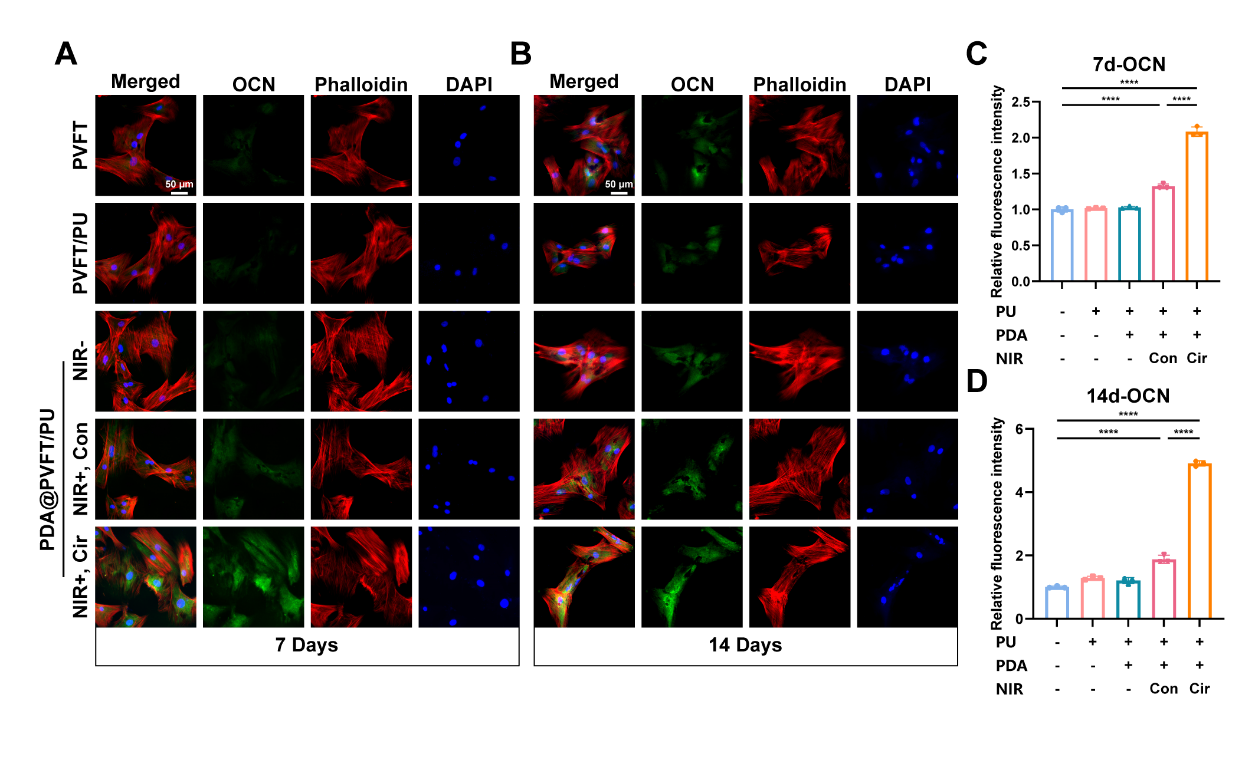


**Supplementary Figure 14.** Representative immunostaining images and quantitative analysis of OCN (green), Phalloidin (red) and cell nuclei (DAPI, blue) of BMSCs cultured in osteogenic differentiation medium for 7 and 14 days (Scale bar = 50 μm). *p < 0.05, **p < 0.01, ***p < 0.001 and ****p <0.0001 (n ≥ 3 per group).


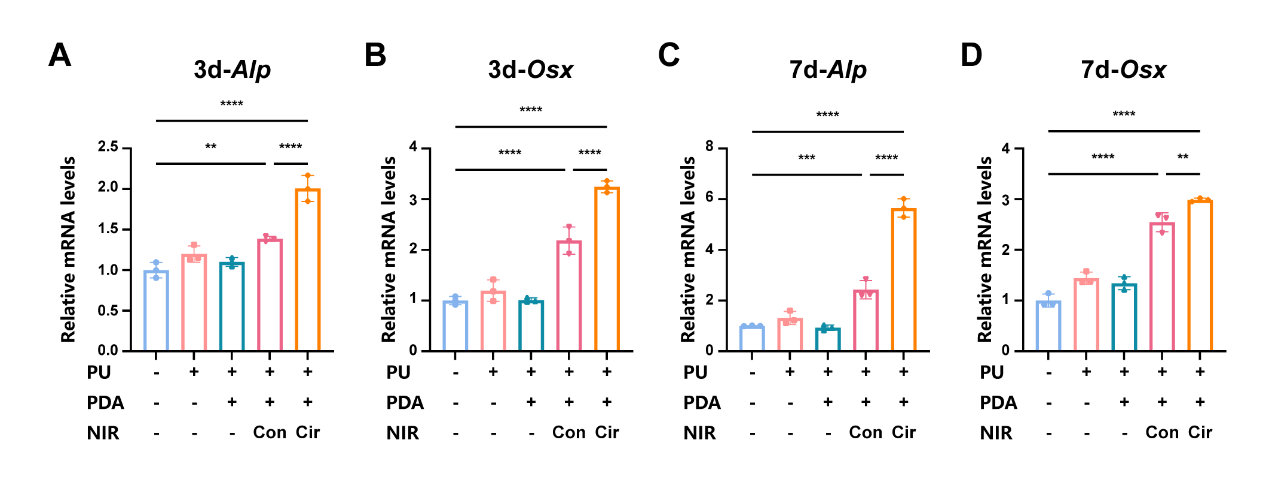


**Supplementary Figure 15.** The mRNA expression levels of osteo-related genes including *Alp* and *Osx* after 3 and 7 days. *p < 0.05, **p < 0.01, ***p < 0.001 and ****p <0.0001 (n ≥ 3 per group).


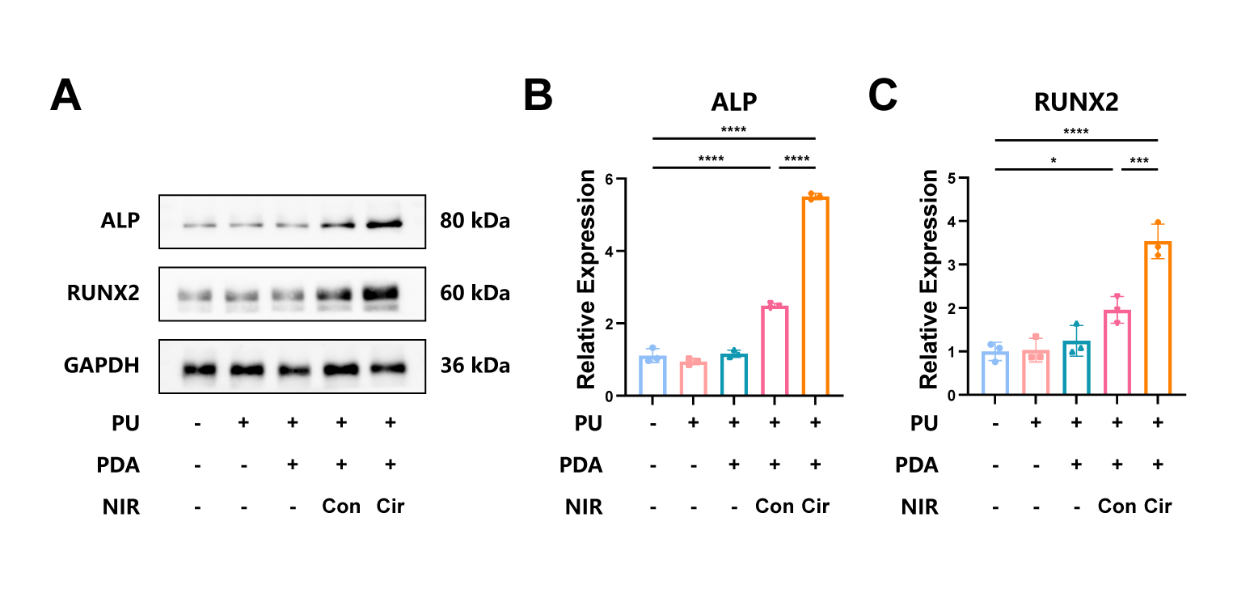


**Supplementary Figure 16.** Representative western blot images and quantitative analysis of osteogenic differentiation-related markers (ALP and RUNX2) of BMSCs cultured for 7 days. *p < 0.05, **p < 0.01, ***p < 0.001 and ****p <0.0001 (n ≥ 3 per group).


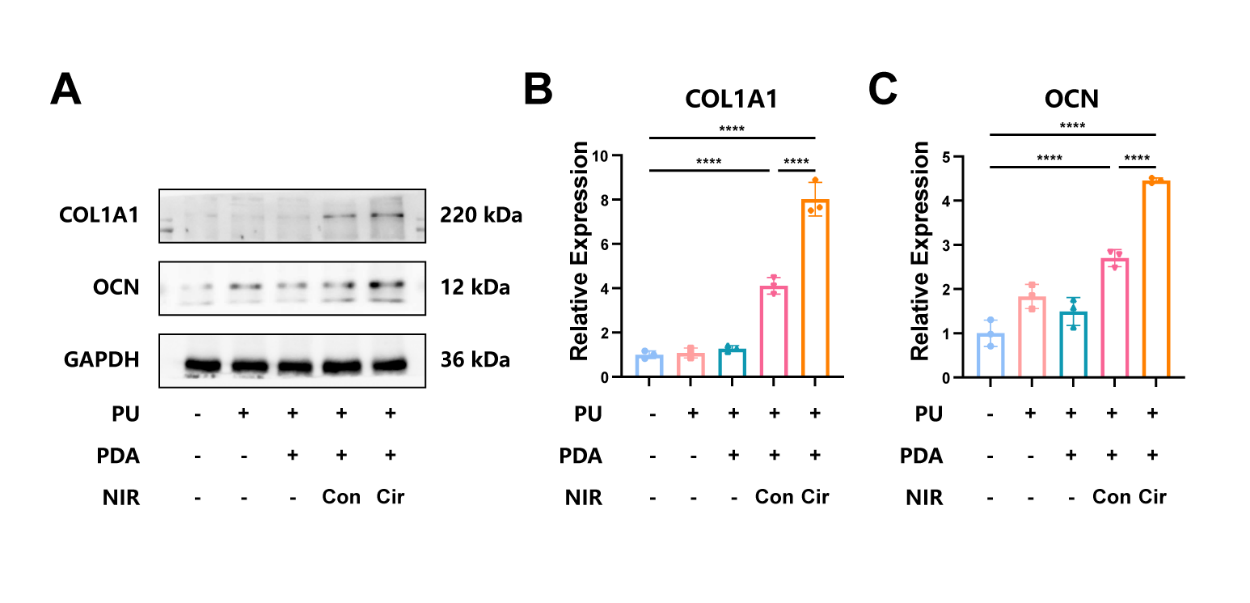


**Supplementary Figure 17.** Representative western blot images and quantitative analysis of matrix mineralization-related markers (COL1A1 and OCN) of BMSCs cultured for 7 days. *p < 0.05, **p < 0.01, ***p < 0.001 and ****p <0.0001 (n ≥ 3 per group).


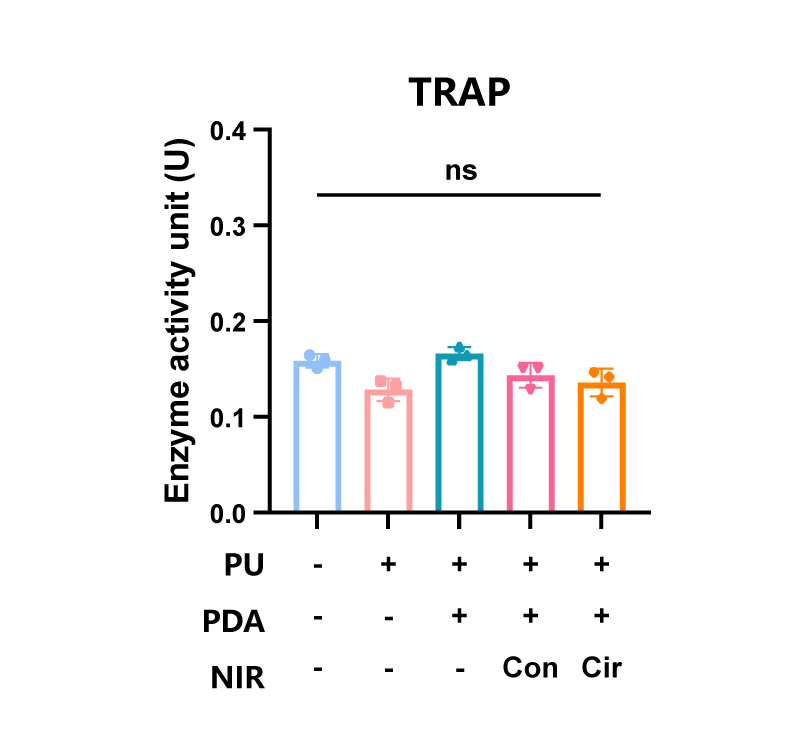


**Supplementary Figure 18.** The TRAP activity of monocytes under continuous and circular NIR irradiation in nanocomposite membranes after 10 days co-culture.


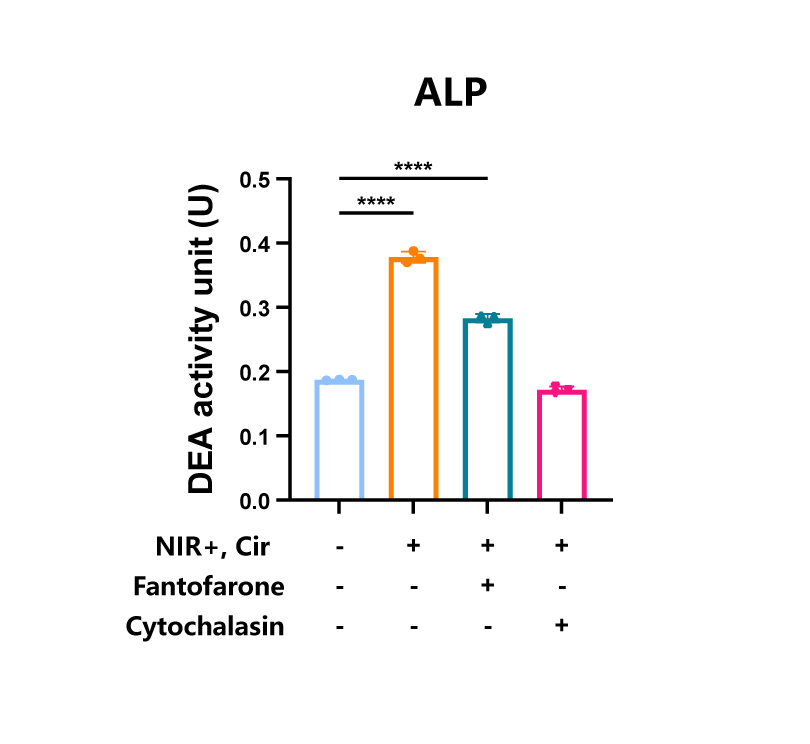


**Supplementary Figure 19.** The 7-day ALP activity of BMSCs with circular NIR irradiation after the addition of calcium channel inhibitors Fantofarone and actin polymerization inhibitors Cytochalasin D. *p < 0.05, **p < 0.01, ***p < 0.001 and ****p <0.0001 (n ≥ 3 per group)


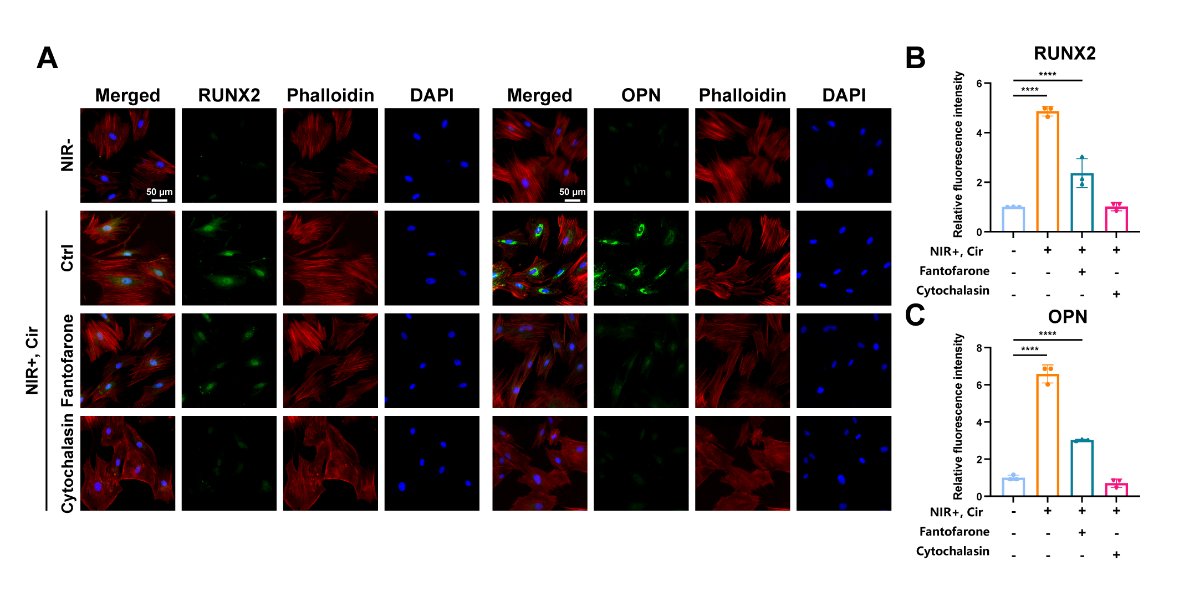


**Supplementary Figure 20.** Representative immunofluorescence images and quantitative analysis of RUNX2 (green), OPN (green), Phalloidin (red) and cell nuclei (DAPI, blue) of BMSCs with 7-day circular NIR irradiation after the addition of calcium channel inhibitors Fantofarone and actin polymerization inhibitors Cytochalasin D (Scale bar = 50 μm). *p < 0.05, **p < 0.01, ***p < 0.001 and ****p <0.0001 (n ≥ 3 per group).


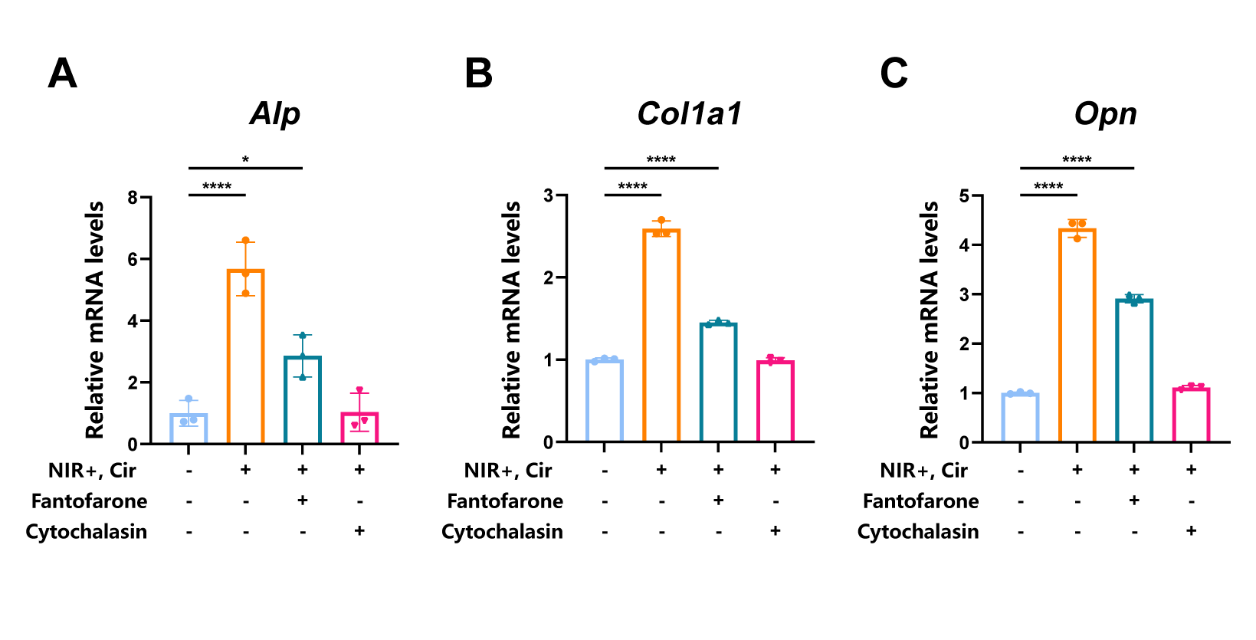


**Supplementary Figure 21.** The mRNA expression levels of osteo-related genes including *Alp*, *Col1a1*and *Opn* after 7 days of culture with the inhibition of calcium channel inhibitors Fantofarone and actin polymerization inhibitors Cytochalasin D. *p < 0.05, **p < 0.01, ***p < 0.001 and ****p <0.0001 (n ≥ 3 per group)


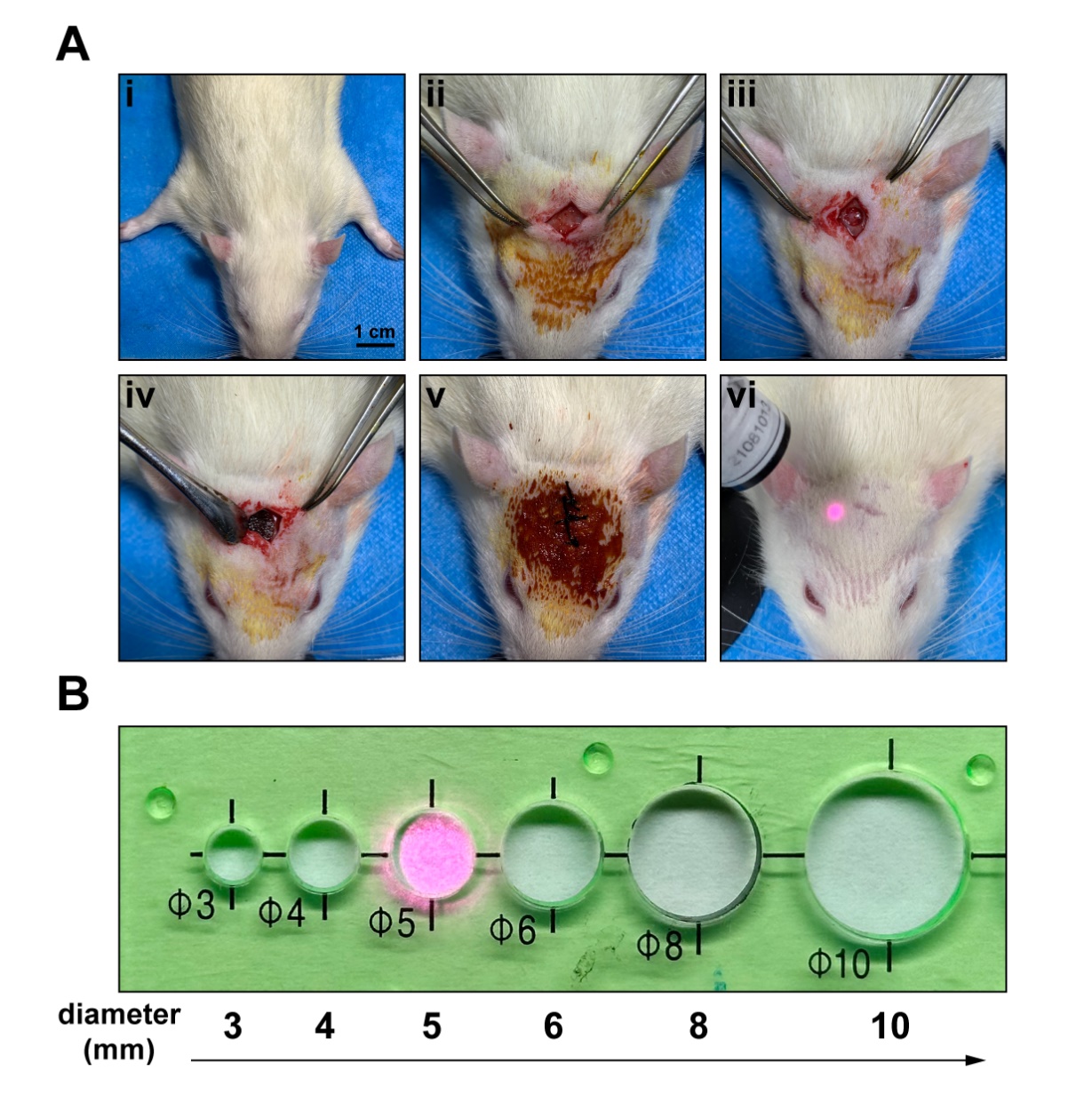


**Supplementary Figure 22.** (A) The skull of Sprague Dawley (SD) rats at the age of 6-8 weeks were used to create the skull defects. (i) and (ii) After anesthetization and disinfection, sagittal incisions were made on the scalp to expose the bilateral skull. (iii) A 5 mm diameter trephine was used to cut circular defects on the skull. (iv) Nanocomposite membranes were implanted into the circular defects. (v) The wounds were sutured and sterilized. (vi) One week later, NIR irradiation treatment was started after suture removal (Scale size = 1 cm). (B) NIR laser transmitting probe produced an irradiated area of 5 mm at the appropriate distance.


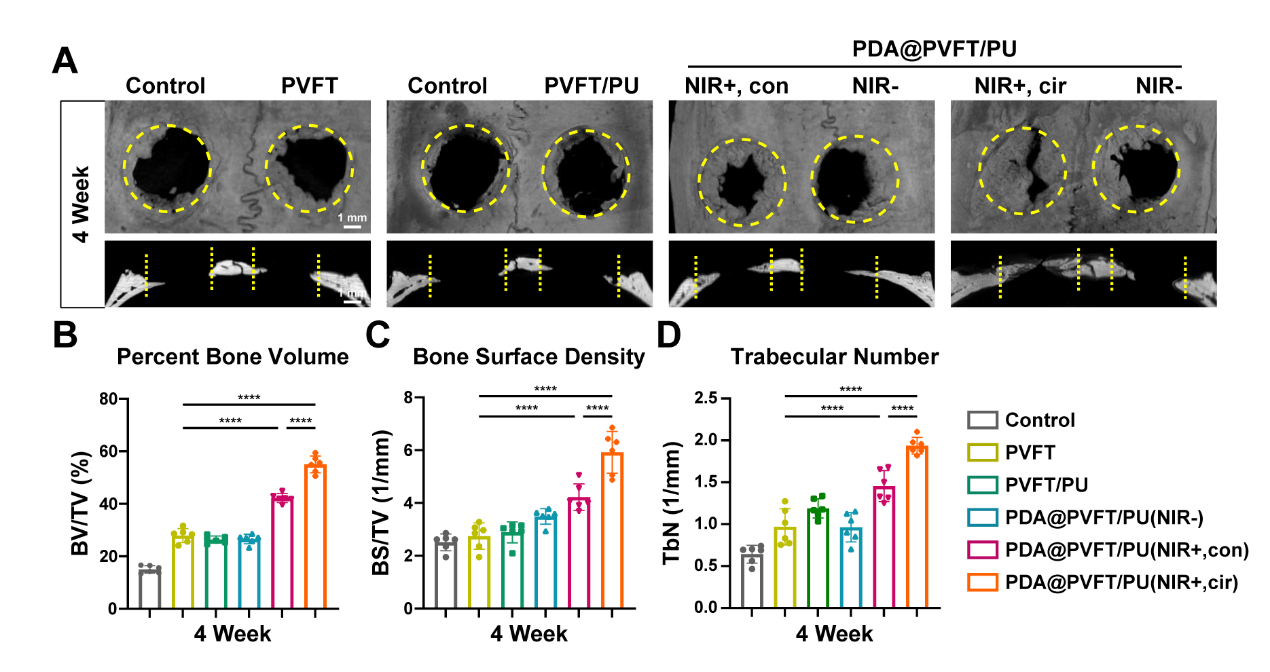


**Supplementary Figure 23.** (A) Representative micro-CT images of critical-sized rat calvarial full-thickness defects after 4-week treatment respectively (Scale bar = 1 mm). Yellow circles and dotted lines denote the boundary between new bone tissue and host bone tissue. (B), (C) and (D) Quantitative analysis of percent bone volume (BV/TV), bone surface density (BS/TV) and Trabecular number (Tb.N). *p < 0.05, **p < 0.01, ***p < 0.001 and ****p <0.0001 (n ≥ 6 per group).


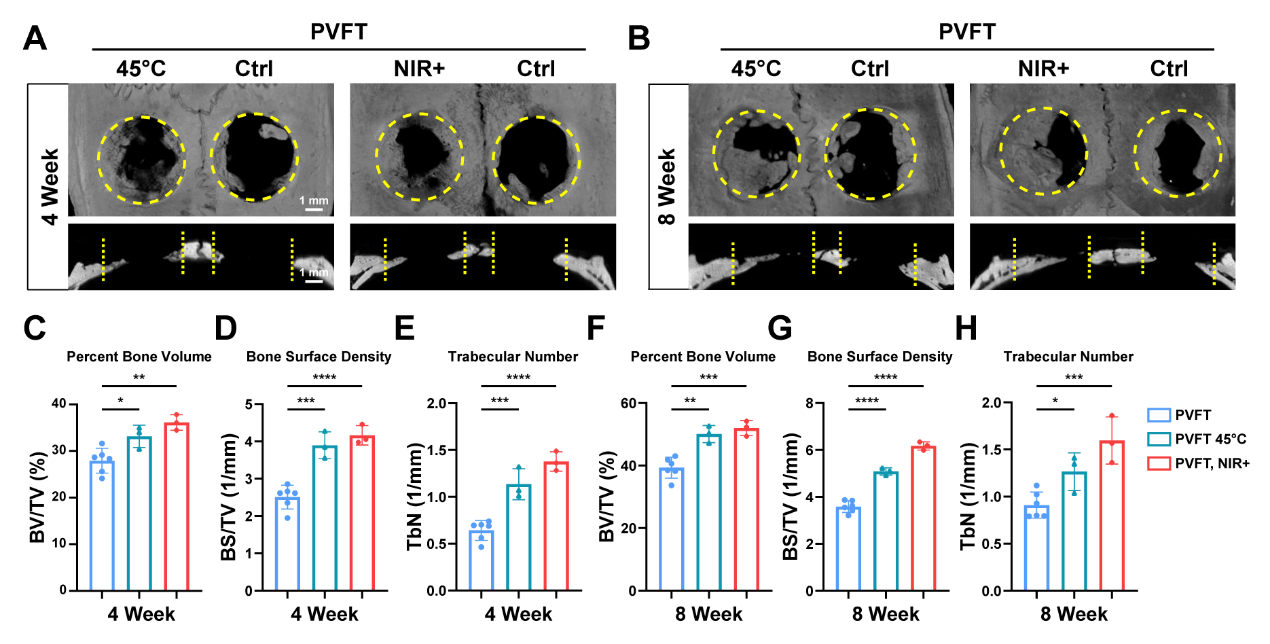


**Supplementary Figure 24.** (A) and (B) Representative micro-CT images of critical-sized rat calvarial full-thickness defects after individual thermotherapy and NIR therapy for 4 and 8 weeks (Scale bar = 1 mm). Yellow circles and dotted lines denote the boundary between new bone tissue and host bone tissue. (C), (D), (E), (F), (G) and (H) Quantitative analysis of percent bone volume (BV/TV), bone surface density (BS/TV) and Trabecular number (Tb.N). *p < 0.05, **p < 0.01, ***p < 0.001 and ****p <0.0001 (n ≥ 3 per group).


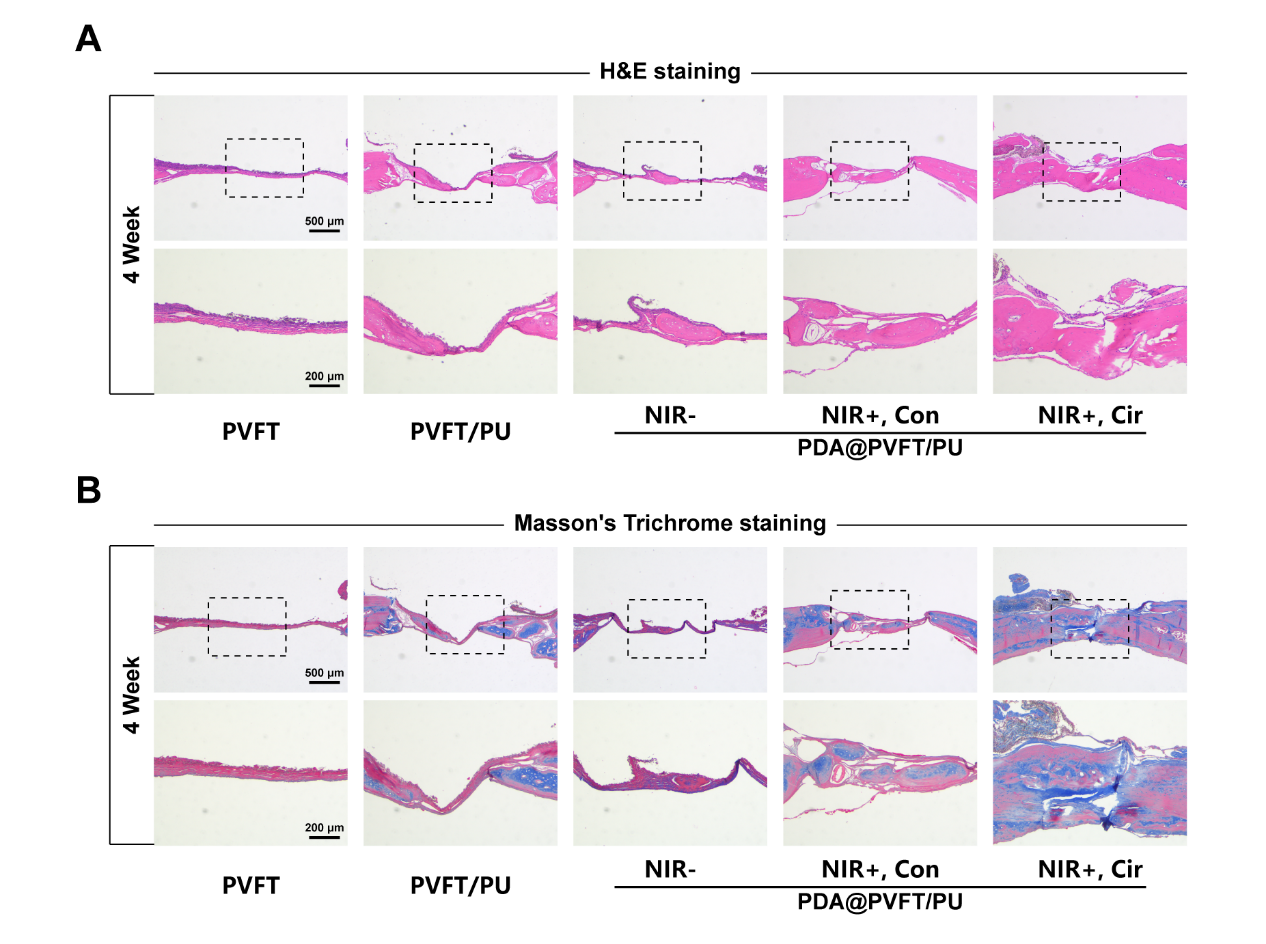


**Supplementary Figure 25.** (A) and (B) H&E staining and Masson’s trichrome staining on histological sections of cranial bone defects at after 4-week irradiation treatment (Scale bar = 500 μm and 200 μm).


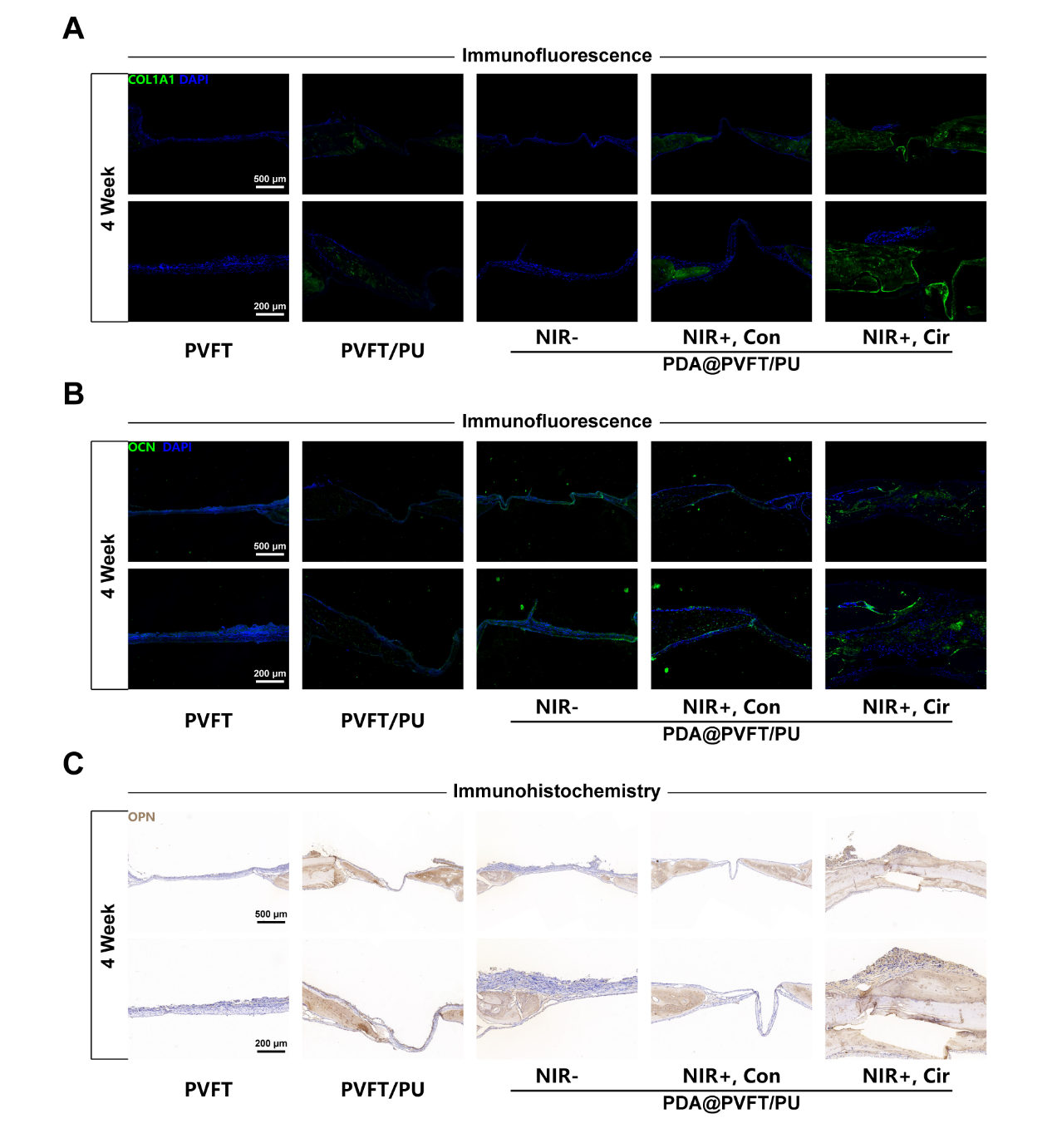


**Supplementary Figure 26.** Representative immunofluorescence images of (A) COL1A1 and (B) OCN and immunohistochemical images of (C) OPN expression levels in histological sections of cranial bone defects after 4-week NIR treatment (Scale bar = 500 μm and 200 μm).


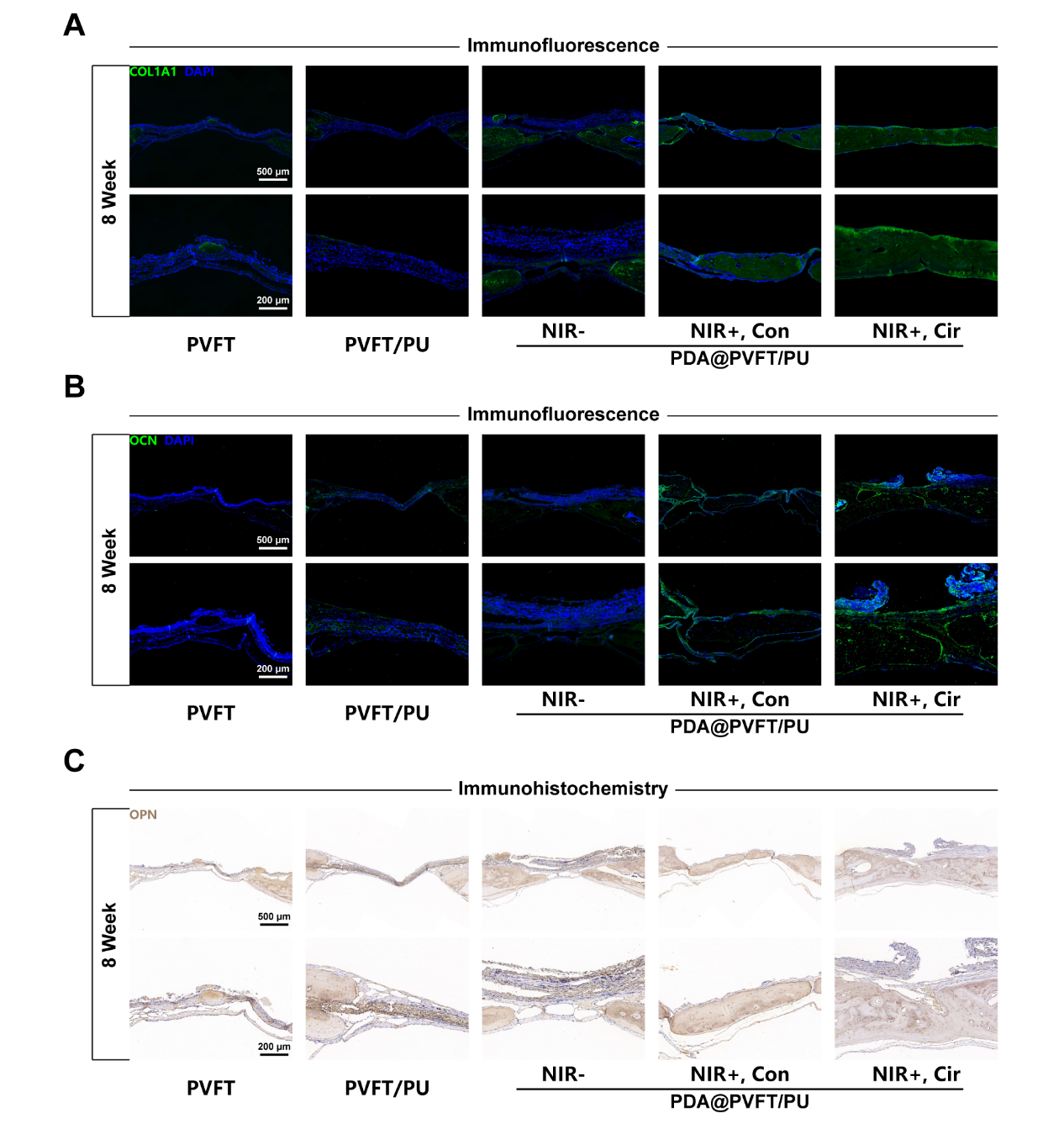


**Supplementary Figure 27.** Representative immunofluorescence images of (A) COL1A1 and (B) OCN and immunohistochemical images of (C) OPN expression levels in histological sections after 8-week NIR treatment (Scale bar = 500 μm and 200 μm).


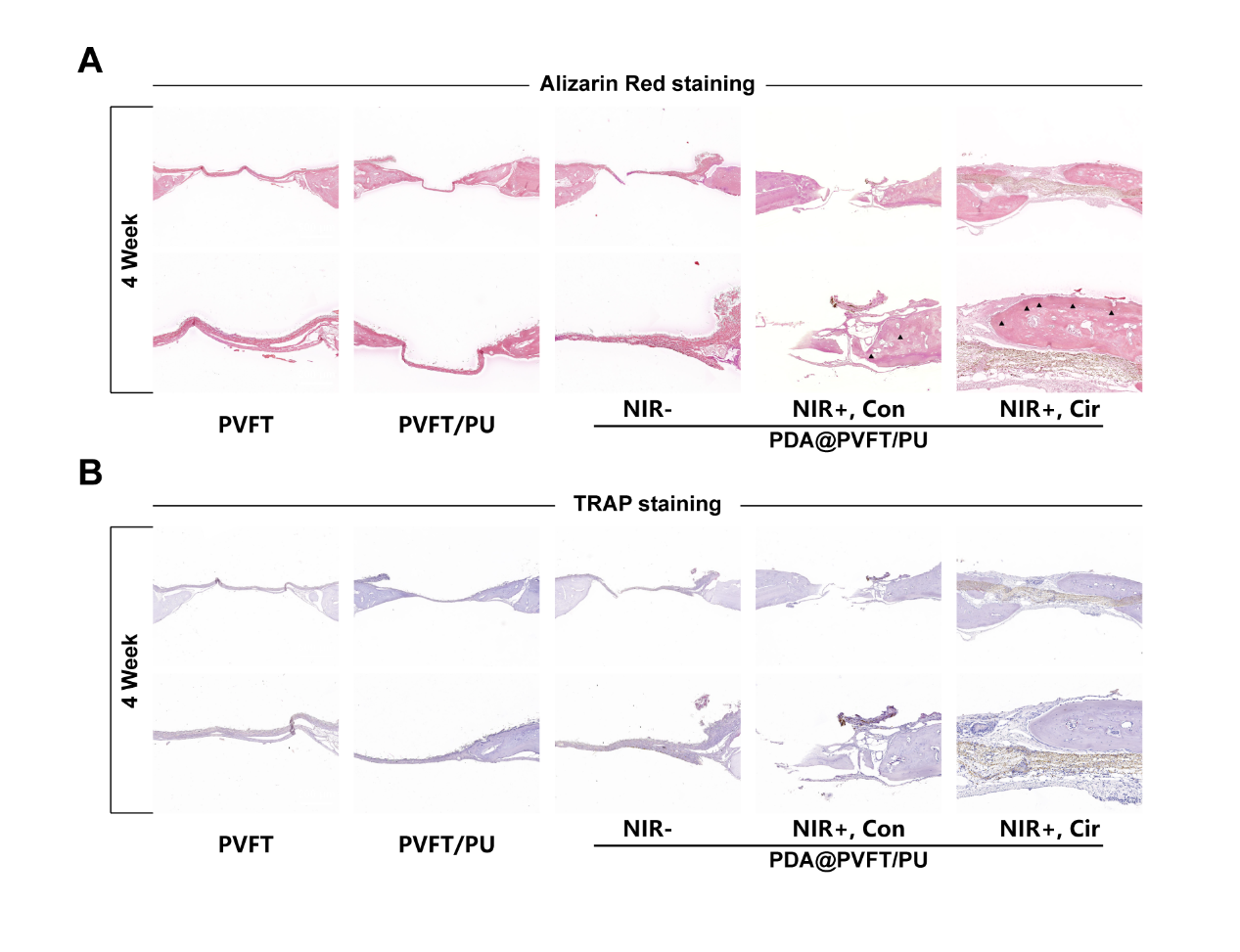


**Supplementary Figure 28.** (A) and (B) Alizarin Red staining and TRAP staining on histological sections of cranial bone defects at after 4-week irradiation treatment. Black triangles denote the mineralized nodules in new bone tissue. (Scale bar = 500 μm and 200 μm).


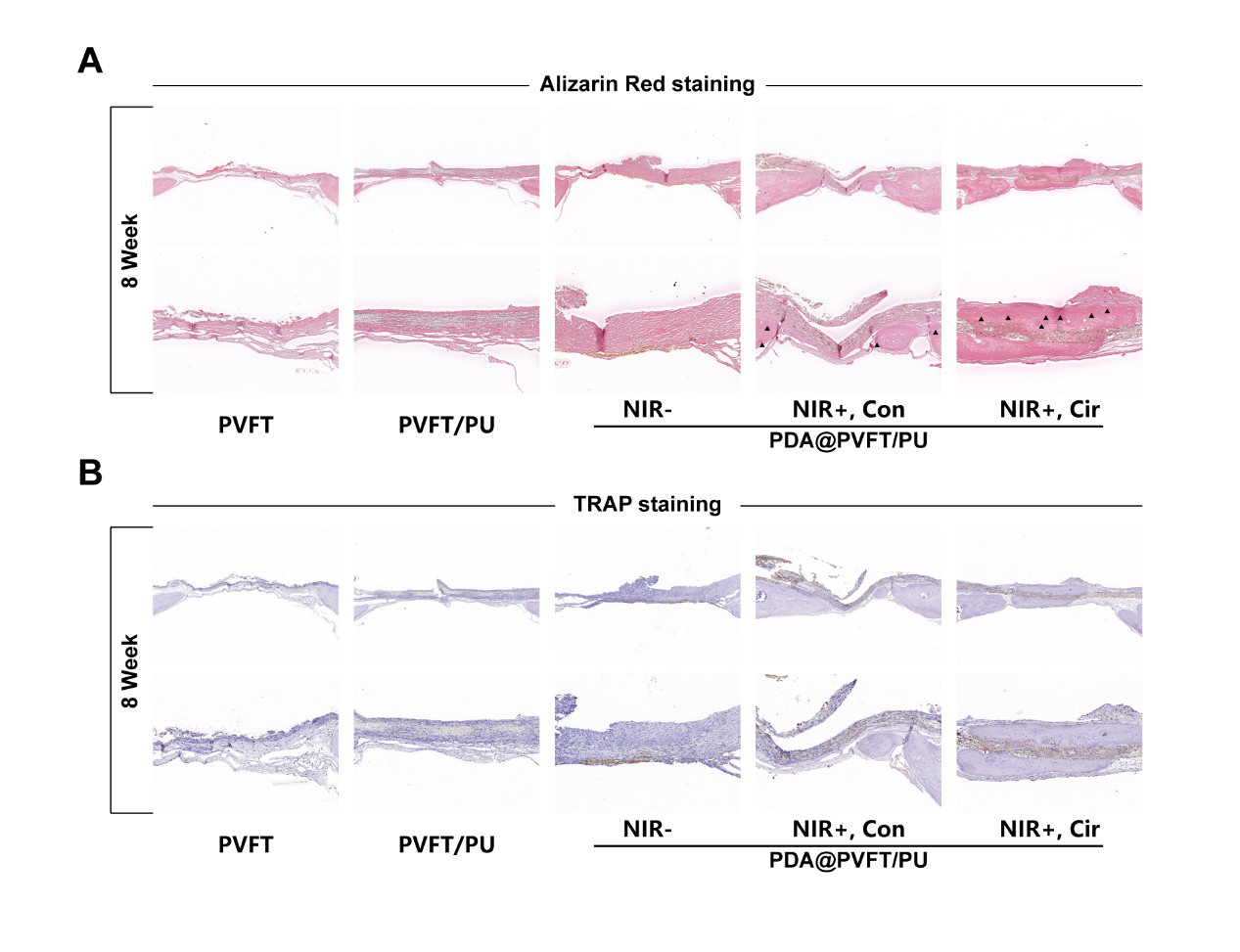


**Supplementary Figure 29.** (A) and (B) Alizarin Red staining and TRAP staining on histological sections of cranial bone defects at after 8-week irradiation treatment. Black triangles denote the mineralized nodules in new bone tissue. (Scale bar = 500 μm and 200 μm).

**Supplementary Tables**

**Table S1.** Materials used in fabrication of PDA@PVFT/PU

| **Materials** | **Vendor or Source** |
| --- | --- |
| N,N-Dimethylformamide (DMF) | Sinopharm Chemical Reagent Co. |
| Polyethylene glycol 2000 (PEG-2000) | Sinopharm Chemical Reagent Co. |
| Phenyl 4,4'-methylenebisisisocyanate (4,4'-MDI) | Sinopharm Chemical Reagent Co. |
| 1,4-Butanediol (BDO) | Sinopharm Chemical Reagent Co. |
| Acetone | Sinopharm Chemical Reagent Co. |
| Sodium hydroxide (NaOH) | Sinopharm Chemical Reagent Co. |
| Polyvinylidene fluoride-Trifluoroethylene [P(VDF-TrFE)] | Arkema |
| Tris-HCl | BioFroxx |
| Dopamine hydrochloride | McLean |

**Table S2**. Primer sequences for qRT-PCR

| **Primer** | **Forward (5'-3')** | **Reverse (5'-3')** |
| --- | --- | --- |
| *r-Gapdh* | CAGTGCCAGCCTCGTCTCAT | AGGGGCCATCCACAGTCTTC |
| *r-Col1a1* | CCTCCCAGAACATCACCTATC | GTGCAGCCATCCACAAGC |
| *r-Runx2* | CCCAGCCACCTTTACCTACA | TATGGAGTGCTGCTGGTCTG |
| *r-Alp* | CCAGCAGGTTTCTCTCTTGG | CTGGGAGTCTCATCCTGAGC |
| *r-Osx* | ACTCATCCCTATGGCTCGTG | GGTAGGGAGCTGGGTTAAGG |

**Table S3.** Information for primary antibodies

| **Primary Antibody** | **Company** | **Cat No.** |
| --- | --- | --- |
| Collagen I/COL1A1 Rabbit pAb | ABclonal, China | A1352 |
| RUNX2 Rabbit pAb | ABclonal, China | A2851 |
| Osteopontin Rabbit pAb | ABclonal, China | A1499 |
| Osteocalcin (BGLAP) Rabbit pAb | ABclonal, China | A6205 |
| Vinculin Rabbit pAb | ABclonal, China | A14193 |
| YAP1 Rabbit pAb | ABclonal, China | A1002 |

**Table S4.** Information for experimental assay kits

| **Name of the kit** | **Company** | **Cat No.** |
| --- | --- | --- |
| Cell Counting Kit-8 (CCK-8) | Beyotime | C0038 |
| Calcein-AM/PI Double Stain Kit | YEASEN | 40747ES76 |
| H5N1Alkaline Phosphatase Assay Kit | Beyotime | P0321S |
| Fluo-4 Calcium Assay Kit | Beyotime | S1061S |
| Mitochondrial membrane potential assay kit | Beyotime | C2006 |
| HiScript III RT SuperMix for qPCR | Vazyme | R323-01 |
| ChamQ Universal SYBR qPCR Master Mix | Vazyme | Q711 |
| Trichrome Stain (Masson) Kit | Sigma-Aldrich | HT15 |
